# Delving into the complexity of hereditary spastic paraplegias: how unexpected phenotypes and inheritance modes are revolutionizing their nosology

**DOI:** 10.1007/s00439-015-1536-7

**Published:** 2015-03-11

**Authors:** Christelle Tesson, Jeanette Koht, Giovanni Stevanin

**Affiliations:** 1 INSERM U1127, CNRS UMR7225, Sorbonne Universités, UPMC Univ Paris 06 UMR_S1127, EPHE, Institut du Cerveau et de la Moelle épinière, CHU Pitié-Salpêtrière, 47 bd de l’Hôpital, 75013 Paris, France; 2 Department of Neurology, Drammen Hospital, Vestre Viken Health Trust, Drammen, Norway; 3 Département de Génétique et Cytogénétique, APHP, Hôpital de la Pitié-Salpêtrière, 75013 Paris, France

## Abstract

Hereditary spastic paraplegias (HSP) are rare neurodegenerative diseases sharing the degeneration of the corticospinal tracts as the main pathological characteristic. They are considered one of the most heterogeneous neurological disorders. All modes of inheritance have been described for the 84 different loci and 67 known causative genes implicated up to now. Recent advances in molecular genetics have revealed clinico-genetic heterogeneity of these disorders including their clinical and genetic overlap with other diseases of the nervous system. The systematic analysis of a large set of genes, including exome sequencing, is unmasking unusual phenotypes or inheritance modes associated with mutations in HSP genes and related genes involved in various neurological diseases. A new nosology may emerge after integration and understanding of these new data to replace the current classification. Collectively, functions of the known genes implicate the disturbance of intracellular membrane dynamics and trafficking as the consequence of alterations of cytoskeletal dynamics, lipid metabolism and organelle structures, which represent in fact a relatively small number of cellular processes that could help to find common curative approaches, which are still lacking.

**Electronic supplementary material:**

The online version of this article (doi:10.1007/s00439-015-1536-7) contains supplementary material, which is available to authorized users.

## Introduction

Hereditary spastic paraplegia (HSP) refers to a group of neurological diseases caused by corticospinal tract degeneration (Tallaksen et al. 2001; Fink 2003, 2013). Approximately, 1 to 10/100,000 people are affected by HSP, depending on the geographical area (Ruano et al. 2014). Patients suffer from the presence of pyramidal signs predominating in lower limbs (LL), which include spasticity (stiff legs) and exaggerated reflexes, associated to muscular weakness that can progress to spastic paralysis of the legs (paraplegia) (Harding 1983; Fink 2003). Pyramidal signs in the upper limbs (UL), as well as distal LL muscle wasting, may appear after long disease durations. Spasticity is usually more severe during gait than at rest. Patients present a swaying, scissor-like, shuffling gait. Age at onset is widely variable, from early childhood to late adulthood. An early sign of spastic paraplegia is the wearing down of the soles of the shoes at the toes and on the inner sides, because of the typical spasticity of adductor muscles and tiptoe gait.

Historically, cases are distinguished as pure or complicated on clinical grounds (Harding 1983), even if recent knowledge of these diseases has demonstrated that this is not always correlated with their genetic bases and can vary between patients in the same family. Pure forms are characterized by pyramidal signs, associated with muscle weakness and bladder dysfunction, but patients may also have decreased vibration sense at ankles or pes cavus. Patients rarely need a wheelchair but may use canes during the disease course, and they have usually, except in some clinico-genetic entities, a normal lifespan. In complicated forms, additional neurological signs are observed, such as cerebellar signs, neuropathy, mental/cognitive impairment, epilepsy, extrapyramidal and retinal signs, as well as extra-neurological signs such as gastroesophageal reflux, cataract and abnormal skin pigmentation. In complex forms, the functional handicap and lifespan will depend on the full clinical picture.

At present, therapeutic options are very limited. For all patients except those with inborn errors of metabolism, rehabilitation therapies with an interdisciplinary approach to maintain autonomy as much as possible, physiotherapy and training are the best treatment options. Regular physical therapy is important to maintain muscle strength and to preserve range of motion and, based on passive tendon stretching, gait and equilibrium rehabilitation. According to the functional repercussion of spasticity, medications such as oral baclofen, intramuscular botulinum toxin or intrathecal baclofen can be of some benefit to patients. Orthopedic options such as special shoes for pes cavus or achilles tendotomy for equinovarus are also proposed to allow a longer autonomous gait. Sphincter disturbances should be investigated by specialists and with a view to possible treatment with anticholinergics, antimuscarinic agents or botulinum toxin injections into the bladder (Fink 2013; Ginsberg et al. 2013). Additional symptoms of complex forms can also be treated, such as parkinsonism with levodopa (Anheim et al. 2009).

## Exclusion diagnosis

There are various acquired and genetic causes that should be ruled out in patients with the symptom of spastic paraplegia without a family history (Table 1). Cerebral and spinal magnetic resonance imaging (MRI) investigations are important to rule out common neurological conditions and structural anomalies (e.g., spinal cord compression). For example, a frontal interhemispheric tumor may manifest as progressive spastic paraplegia with sphincter disturbances before other signs such as cognitive deterioration, headache or visual troubles appear. Disease progression, age at onset, additional symptoms and results from other supplementary investigations such as cerebrospinal fluid (CSF) analyses, blood biochemistry and serology, electroneuromyography and ophthalmological examination can give important clues to the diagnosis (Table 1). All these investigations will first exclude acquired causes of spastic paraplegia but will subsequently help with the diagnostic workflow to find the correct genetic diagnosis. Some apparently sporadic cases are in fact masked familial diseases. The absence of a family history in neurogenetic disorders is frequent in clinical practice and several explanations for apparent isolation are reduced penetrance, age-dependent penetrance, variable expressivity, de novo mutation, early death of the transmitting parent or underdiagnosis in pure dominant forms with mild symptoms, autosomal recessive inheritance in small kindreds or, more rarely, X-linked inheritance in affected men. Among other inherited neurogenetic conditions that must be ruled out are leukodystrophies, in the absence of inflammation but in the presence of MRI abnormalities. Biochemical analyses in serum and/or CSF can suggest neurometabolic diseases. Finally, dopa-responsive dystonias (DRD) are a group of autosomal dominant or recessive diseases, which may present with spasticity and can mimic HSP. The dystonic toe is well known and can be misdiagnosed as extensor plantar reflex (Furukawa et al. 2001). Diurnal fluctuations and high and sustained sensibility to levodopa are characteristic of DRD.

**Table 1:** List of the most important differential diagnoses to hereditary spastic paraplegia with suggested supplementary investigations

Type of disease	Disease	Investigations	Characteristics other than spasticity and Babinski sign^b^
Structural anomalies and trauma	Arnold–Chiari malformation	Brain and spine MRI	Ataxia, dizziness, unsteadiness
	Tumor	Brain and spine MRI	Headache if brain tumor, other focal symptoms
	Spinal cord vascular malformation	MRI/spinal angiography	Fluctuating symptoms/sudden onset
	Vertebral disorders with myelopathy	Spine MRI	Sensory symptoms, pain
	Spinal cord injury	Spine MRI	Sudden onset, trauma
Inflammatory	Primary progressive or relapsing-remitting multiple sclerosis	Brain and spine MRI, CSF investigations including immunoelectrophoresis (evoked responses)	Symptoms from different topographic regions
Neurodegenerative	Spinocerebellar ataxias	Genetic screening, brain MRI	Ataxia
	Amyotrophic lateral sclerosis (ALS) and primary lateral sclerosis (PLS)	Spine and brain MRI, neurography, electromyography, CSF investigations	Often bulbar signs and rapid progression, weakness, increased reflexes. In ALS; upper and lower motor neuron signs
Acquired	Diplegic cerebral palsy (Little disease)	Brain MRI, antenatal, birth or postnatal history	Non-progressive
Infectious	Neurosyphilis	Syphilis serology/CSF investigations	Acute/subacute, and chronic, laboratory findings, often peripheral nervous system findings
	HTLV-1 infection (tropical spastic paraparesis)	Serum/CSF HTLV-1 antibodies	Subacute onset, laboratory findings
	Acquired immune deficiency syndrome (AIDS)	HIV test	Subacute onset, laboratory findings
	Neuroborreliosis	Serology/CSF investigations	Subacute onset, laboratory findings and/or symptoms from other topographic regions other than upper motor neuron
Metabolic^a^	Leukodystrophies		
	X-linked adrenoleukodystrophies	Brain MRI, measurement of very long-chain fatty acids in plasma	Neuropathy, cognitive decline, white matter changes
	Metachromatic leukodystrophy (late-onset forms)	Brain MRI, arylsulphatase A dosage	Neuropathy, behavioral signs and regression
	Hereditary CNS demyelinating disease		
	Krabbe leukodystrophy (late-onset forms)	Brain MRI, galactocerebrosidase deficiency	Neuropathy, regression
	Pelizaeus–Merzbacher disease	Brain MRI	Nystagmus, ataxia, developmental delay
	Canavan disease	Brain MRI, excessive urinary NAA excretion	Blindness, severe mental defect, megalocephaly
	Leukoencephalopathy with vanishing white matter	Brain MRI	Also known as childhood ataxia with central nervous system hypomyelination (CACH) or vanishing white matter disease
	Alexander disease	Brain MRI	Seizures, megalencephaly, developmental delay; In older patients, bulbar or pseudobulbar signs
	Sjögren–Larsson syndrome (progressive forms)	Brain MRI, low fatty aldehyde dehydrogenase activity	Ichthyosis, mental retardation, macular dystrophy and leukoencephalopathy
	Refsum disease	Brain MRI, accumulation of an unusual branched-chain fatty acid, phytanic acid, in blood and tissues	Retinitis pigmentosa, peripheral neuropathy, cerebellar ataxia
	Cerebrotendinous xanthomatosis	Brain MRI, deposits of cholesterol and cholestanol in virtually every tissue	Cerebellar ataxia beginning after puberty and pseudobulbar phase leading to premature death
	Subacute combined degeneration of the cord and anemia/Lichtheim disease	Blood cell counts, vitamin B12 dosage, Schilling test (vitamin B12 absorption)	Neuropathy, anemia
	Amino acid disorders, e.g., Arginase deficiency	Plasma arginine level, aminoaciduria, genetic screening	Developmental delay, intellectual disability, seizures, tremor, ataxia, fluctuating symptoms
	Mitochondrial disorders	Lactate and pyruvate levels in blood and CSF, muscle biopsy	Dependent of the heteroplasmy levels, symptoms from different organs (multisystemic)
	Abetalipoproteinemia	Lipoprotein electrophoresis	Neuropathy, ataxia
	Vitamin E deficiency	Serum vitamin E level	Often with neuropathy and ataxia
	Dystonia (including dopa-responsive dystonias)	l-Dopa trial, neurotransmitter investigations in CSF, CSF/serum glucose ratio for GLUT1 deficiency, genetic screening (heterozygous *GCH1* mutations and to a lesser extend biallelic *TH* and *SPR* mutations for dopa-responsive forms, heterozygous *SLC2A1* mutations in DYT9/GLUT1)	Early-onset, fluctuating symptoms/diurnal variation
Brain metal accumulation disorders	Wilson disease (progressive forms)	Brain MRI, serum copper and ceruloplasmin, 24-h urine copper; liver tissue biopsy	Basal ganglia dysfunction symptoms
	Neurodegeneration with brain iron accumulation (NBIA)	Brain MRI, genetic screening (particularly *PANK2, COASY, PLA2G6, ATP13A2, WDR45* and allelic HSP forms; *FA2H/SPG35, C19orf12/SPG43*)	Early onset and rapid progression particularly in PANK2 mutated patients, dystonia, central region of hyperintensity in the globus pallidus with surrounding hypointensity on T2-weighted images (“eye-of-the-tiger sign”)
Toxic causes	Neurolathyrism	Epidemical context, Africa	Ingestion of certain vegetables of the genus *Lathyrus* (peas…), subacute
	Konzo	Epidemical context, Africa	Improper preparation and ingestion of cassava roots, onset less than one week later, non-progressive
	Heavy metals (copper, manganese, lead)	Brain and spine MRI, CSF investigations	Diffuse clinical picture, exposure context to heavy metals

*CNS* central nervous system, *CSF* cerebrospinal fluid, *HIV* human immunodeficiency virus, *HTLV-1* Human T cell leukemia/lymphoma virus type 1, *MRI* magnetic resonance imaging, *NAA* *N*-acetylaspartic acid

^a^The list is not complete, but the main groups with the most important subgroups are mentioned

^b^Extensor response of the cutaneous plantar reflex

The exploration of rare genetic disorders is an important issue since some diseases associated with spasticity are treatable. In particular, spastic paraparesis can be one of the multiple presentations of inborn errors of metabolism in children and adults and in some cases the symptom spastic paraparesis remains the only symptom for years; therefore, these metabolic causes should be included in the general diagnostic approach to sporadic spastic paraparesis due to treatment options (e.g., diet for argininemia, biotin in biotinidase deficiency) (Tanyel and Mancano 1997; Sedel et al. 2007) (www.treatable-id.org).

## Genetic aspects of HSP

Genetic analysis of HSP genes can be performed when, according to the clinical symptoms and signs, other important causes have been excluded. HSP genes are denoted Spastic Paraplegia Gene followed by a number according to their order of discovery (SPGn). Up to now, the clinical phenotype and age at onset were critical to prioritize molecular testing because of the heterogeneity of these diseases at the clinical and genetic levels (Supplementary Fig. S1). More than 25 novel causative genes have been reported in 2013–2014 due to next-generation sequencing methods, making this genetic workflow time-consuming (Martin et al. 2013; Oates et al. 2013; Boukhris et al. 2013; Landouré et al. 2013; Novarino et al. 2014; Dor et al. 2014; Esteves et al. 2014; Crow et al. 2014), even if there are some genes that are still more frequent than others and may be analyzed first, such as *SPAST* (SPG4) and *KIAA1840* (SPG11) (see below). All classical modes of transmission can be found and there are at least 67 genes that, when mutated, can account for these diseases (Table 2) to which can be added additional genes for which spasticity can be present as part of the clinical presentation (Supplementary Table 1).

**Table 2:** HSP genes and their associated phenotypes

SPG no (HUGO) (inheritance)	Chr Gene (OMIM no)	Age at onset (y)	Pure (P) or Complex (C) forms	Associated clinical features (OMIM no) Functional tests, biomarkers	MRI features	References	Allelic disorders (OMIM no)^b^
SPG1 (X-linked)	Xq28 *L1CAM* (308840)	Congenital	C	MASA syndrome (Mental retardation Aphasia Shuffling gait and Adducted thumb) or CRASH syndrome (Corpus callosum hypoplasia Retardation Adducted thumb Spastic paraplegia and Hydrocephalus) (303350)	Agenesis of the corpus callosum and hydrocephalus	Rosenthal et al. (1992) Jouet et al. (1994)	Hydrocephaly (307000); Corpus callosum agenesis (304100)
SPG2 (X-linked)	Xq22.2 *PLP1* (300401)	Variable	P or C	Spastic paraplegia with nystagmus, cerebellar dysfunction, hypotonia, MR and sometimes dementia or seizures (312920)	WMH	Cremers et al. (1987) Saugier-Veber et al. (1994)	Pelizaeus–Merzbacher disease (312080)
SPG3/SPG3A (AD/AR)	14q22.1 *ATL1* (606439)	<1 to 51 (mainly <10)	P (C)	Pure form, rarely with axonal neuropathy or amyotrophy, incomplete penetrance (182600)	Normal (one family with late onset and TCC)	Zhao et al. (2001)	Hereditary sensory neuropathy type ID, AD (613708)
SPG4 (AD)	2p22.3 *SPAST* (604277)	1–80	P (C)	Pure, rarely with cognitive impairment or neuropathy; epilepsy, ataxia and ALS in one family, incomplete penetrance (182601)	Normal (WMH in one family)	Hazan et al. (1999)	
SPG5/SPG5A (AR)	8q12.3 *CYP7B1* (603711)	4–47	P or C	Pure or with cerebellar signs, nystagmus, cognitive impairment and amyotrophy (270800) 27-hydroxy-cholesterol accumulation in blood and CSF	Normal (rarely WMH)	Tsaousidou et al. (2008) Schüle et al. (2010)	Bile acid synthesis defect (613812); Sensory ataxia
SPG6 (AD)	15q11.2 *NIPA1* (608145)	8–37	P (C)	Pure, rarely with neuropathy or epilepsy or memory impairment (600363)	Normal	Rainier et al. (2003)	
SPG7 (AR)	16q24.3 *SPG7* (602783)	4–42	P or C	Pure or with optic neuropathy or cerebellar ataxia (607259) Mito DNA deletions, defects in Mito respiration	Normal or cerebellar atrophy	Casari et al. (1998) Wedding et al. (2014)	Optic neuropathy, AD; Late-onset ataxia susceptibility, AD
SPG8 (AD)	8q24.13 *KIAA0196* (610657)	10–60	P (C)	Rarely complex with neuropathy (603563) Decreased Cho & Cr/NAA peak at PMRS	Normal, or few white matter abnormalities and atrophy of the thoracic spinal cord	Valdmanis et al. (2007) Wang et al. (2014)	Ritscher–Schinzel syndrome, AR (220210)
SPG9 (AD)	10q23.3-q24.2-	1–30	C	Bilateral cataracts, gastroesophageal reflux, neuropathy, amyotrophy (601162)	Normal (atrophy limited to spinal cord)	Seri et al. (1999)	
SPG10 (AD)	12q13.3 *KIF5A* (602821)	2–51	P or C	Pure or with neuropathy (Silver syndrome) (604187)	Normal	Reid et al. (2002)	
SPG11 (AR)	15q21.1 *KIAA1840* (610844)	<1 to 33	P or C	Mostly complex with cognitive decline, neuropathy, retinopathy (Kjellin syndrome) and cerebellar signs (604360)	TCC, WMH and cerebellar atrophy	Stevanin et al. (2007b)	Juvenile amyotrophic lateral sclerosis (ALS-5), Orlacchio et al. (2010)
SPG12 (AD)	19q13.32 *RTN2* (603183)	7–24	P	Pure (604805)	Normal or with WMH	Montenegro et al. (2012)	
SPG13 (AD)	2q33.1 *HSPD1* (118190)	17–68	P	Pure (605280)	Normal	Hansen et al. (2002)	Hypomyelinating leukodystrophy type 4, AR (612233)
SPG14 (AR)	3q27-q28	~30	C	Distal motor neuropathy, mild MR, visual agnosia, and memory deficiency (605229)	Normal	Vazza et al. (2000)	
SPG15 (AR)	14q24.1 *ZFYVE26* (612012)	4–19	P or C	Mostly complex with cognitive decline, neuropathy, retinopathy (Kjellin syndrome) and cerebellar signs (270700)	TCC, WMH and cerebellar atrophy	Hanein et al. (2008)	
SPG16 (X-linked)	Xq11.2	Early infancy	P or C	Pure or complex with quadriplegia, motor aphasia, mild MR, and bowel and bladder dysfunction (300266)	Delayed myelination	Steinmüller et al. (1997) Tamagaki et al. (2000)	
SPG17 (AD)	11q12.3 *BSCL2* (606158)	2–60	C	Silver syndrome: neuropathy, amyotrophy (270685)	Normal	Magré et al. (2001); Windpassinger et al. (2004)	Congenital lipodystrophy type 2, AR (260700); Hereditary motor neuropathy type VA, AD (600794); Progressive encephalopathy, AR (615924)
SPG18 (AR)	8p11.23 *ERLIN2* (611605)	<2	C	ID and contractures (611225)	Normal	Yıldırım et al. (2011)	Juvenile primary lateral sclerosis, AR
SPG19 (AD)	9q33-q34	36–55	P	Pure (607152)	Normal	Valente et al. (2002)	
SPG20 (AR)	13q12.3 *SPG20/KIAA0610* (607111)	Infancy	C	Troyer Syndrome: dysarthria, distal amyotrophy in hands and feet, cerebellar signs, mild ID and skeletal abnormalities (short stature) (275900)	WMH	Patel et al. (2002)	
SPG21 (AR)	15q22.31 *SPG21/ACP33* (608181)	Adulthood	C	Mast syndrome: speech decline leading to akinetic mutism, personality disturbances, psychotic episodes, cognitive decline and cerebellar dysfunction (incoordination and dysdiadochokinesia). For a Japanese family: cognitive decline and apraxia (248900)	TCC, WMH and cerebellar atrophy	Simpson et al. (2003)	
SPG22 (X-linked)	Xq13.2 *SLC16A2* (300095)	Early infancy	C	Allan–Herndon–Dudley syndrome: spastic quadriplegia, severe MR, central hypotonia, muscle hypoplasia, dystonia, ataxia (300523) Abnormal relative concentrations of circulating iodothyronines	Normal or most often delayed myelination with sometimes TCC and mild cortical atrophy	Dumitrescu et al. (2004) Schwartz et al. (2005)	
SPG23 (AR)	1q24-q32	Infancy	C	Lison syndrome: abnormal skin and hair pigmentation, ± dysmorphisms, skeletal deformities, MR or sensorimotor neuropathy (270750)	Normal or slight enlargement of the ventricles with ± microcephaly	Blumen et al. (2003)	
SPG24 (AR)	13q14	Infancy	P	Pure (607584)	Normal	Hodgkinson et al. (2002)	
SPG25 (AR)	6q23-24.1	30–46	C	Mild sensorimotor neuropathy (608220)	Spinal disc herniation with minor spondylosis	Zortea et al. (2002)	
SPG26 (AR)	12q13.3 *B4GALNT1* (601873)	2–19	C	ID, cerebellar ataxia, peripheral neuropathy, and one family presents behavioral problems (609195) Decreased GM2 and increased GM3 in fibroblasts. Low testosterone level in men	Normal or after long disease duration cortical and subcortical atrophy and/or WMH	Boukhris et al. (2013) Harlalka et al. (2013)	
SPG27 (AR)	10q22.1-q24.1	P: 25–45 C: 2–7	P or C	Pure or with sensorimotor polyneuropathy and sometimes with MR, cerebellar signs and skeletal abnormalities (609041)	Normal or mild cortical and cerebellar atrophy	Meijer et al. (2004) Ribai et al. (2006)	
SPG28 (AR)	14q22.1 *DDHD1* (614603)	7–15	P or C	Pure or with cerebellar oculomotor disturbances or axonal neuropathy (609340) Ventricular lactate accumulation and reduction of PCr/Pi ratio in muscles	Normal	Tesson et al. (2012) Liguori et al. (2014)	
SPG29 (AD)	1p31.1-21.1	Infancy	C	Neonatal hyperbilirubinemia, hearing impairment due to auditory neuropathy and persistent vomiting due to hiatal hernia (609727)	Normal	Orlacchio et al. (2005)	
SPG30 (AR)	2q37.3 *KIF1A* (601255)	10–39	P or C	Pure or with sensory neuropathy and cerebellar ataxia (610357)	Normal or mild cerebellar atrophy	Erlich et al. (2011); Klebe et al. (2012b)	Complex MR with axial hypotonia, spasticity and cerebellar atrophy, AD (614255); Sensory and autonomic neuropathy, AR (614213)
SPG31 (AD)	2p11.2 *REEP1* (609139)	Variable	P or C	Pure or sometimes complex with neuropathy (610250)	Normal	Züchner et al. (2006)	Distal hereditary motor neuropathy type VB, AD (614751)
SPG32 (AR)	14q12-q21	6–7	C	Mild MR (611252)	Cerebellar atrophy and pontine dysraphia, moderate TCC	Stevanin et al. (2007a)	
SPG33 (AD)	10q24.2 *ZFYVE27* (610244)	42–50	P	Pure (610248)	ND	Mannan et al. (2006)	
SPG34 (X-linked)	Xq24-q25	16–25	P	Pure (300750)	ND	Macedo-Souza et al. (2008)	
SPG35 (AR)	16q23.1 *FA2H* (611026)	2–17 one family with late onset	C	Dystonia, LL amyotrophy, seizures, cerebellar signs, cognitive decline and optic atrophy (612319) Reduced hydroxylated fatty acid sphingomyelin in fibroblasts and erythrocytes	Leukodystrophy, hypointensities of globus pallidus, TCC and cerebellar atrophy	Edvardson et al. (2008) Dan et al. (2011)	Leukodystrophy/NBIA, AR
SPG36 (AD)	12q23-24	14–33	C	Peripheral sensorimotor neuropathy (613096)	Normal	Schüle et al. (2009a)	
SPG37 (AD)	8p21.1-q13.3	8–60	P	Pure (611945)	Normal	Hanein et al. (2007)	
SPG38 (AD)	4p16-p15	16–19	P	Clinical features similar to SPG4 (612335)	ND	Orlacchio et al. (2008)	
SPG39 (AR)	19p13.2 *PNPLA6* (603197)	Infancy, adolescence	C	Muscle wasting and motor axonopathy of the LL and UL (612020)	Normal	Rainier et al. (2008); Synofzik et al. (2014)	Boucher-Neuhauser syndrome (215470); Gordon Holmes syndrome; Spastic ataxia
SPG41 (AD)	11p14.1-11p.2	Mean 17 ± 3	P	Pure (613364)	Normal	Zhao et al. (2008)	
SPG42 (AD)	3q25.31 *SLC33A1* (603690)	4–42	P	Pure (612539)	Normal	Lin et al. (2008)	Congenital cataracts, hearing loss and neurodegeneration, AR (614482)
SPG43 (AR)	19p13.11-q12 *C19orf12* (614297)	7–12	C	Neuropathy and severe atrophy and decreased sensation in the arms and legs (615043)	Normal	Landouré et al. (2013)	NBIA4 (614298); Pallido-pyramidal syndrome
SPG44 (AR)	1q42.13 *GJC2* (608803)	1st or 2nd decade	C	Dysarthria, cerebellar ataxia, mental impairment (613206) Reduced Cho/NAA and Cho/Cr ratios	WMH	Uhlenberg et al. (2004) Orthmann-Murphy et al. (2009)	Pelizaeus–Merzbacher-like hypomyelinating leukodystrophy (608804); Hereditary lymphedema, AD (613480)
SPG45 (AR)	10q24.3-q25.1	Infancy	C	MR and ocular signs (613162)	ND	Dursun et al. (2009)	
SPG46 (AR)	9p13.3 *GBA2* (609471)	1–16	C	Cerebellar ataxia, cataract and mental impairment, infertility in males (614409) GBA2 activity abolished in lymphoblasts and leukocytes	TCC, cerebral and cerebellar atrophy	Martin et al. (2013)	Spastic ataxia
SPG47 (AR)	1p13.2 *AP4B1* (607245)	Birth	C	Severe ID, absent speech, shy character, stereotypic laughter, muscular hypotonia, microcephaly, foot deformity, decreased muscle mass and growth retardation (614066)	Periventricular WMH and TCC	Abou Jamra et al. (2011) Bauer et al. (2012)	
SPG48 (AR)	7p22.1 *AP5Z1* (613653)	2–50	P or C	Pure or with cognitive impairment or MR (613647)	Normal or TCC and WMH	Słabicki et al. (2010)	
SPG49^a^ (denoted SPG56 by OMIM) (AR)	4q25 *CYP2U1* ^a^ (615030)	<1–8	P or C	Mental impairment, dysarthria, dystonia and infraclinical axonal neuropathy (615030)	Normal or TCC, WMH and basal ganglion calcifications	Tesson et al. (2012)	
SPG50 (AR)	7q22.1 *AP4M1* (602292)	Infancy	C	Tetraplegic cerebral palsy with MR (612936)	WMH and cerebellar atrophy	Verkerk et al. (2009)	
SPG51 (AR)	15q21.2 *AP4E1* (607244)	Infancy	C	Similar to SPG47 (613744)		Abou Jamra et al. (2011)	
SPG52 (AR)	14q12 *AP4S1* (607243)	Infancy	C	Similar to SPG47 (614067)		Abou Jamra et al. (2011)	
SPG53 (AR)	8p22 *VPS37A* (609927)	1–2	C	Developmental and motor delay, delays in cognition and speech, marked kyphosis (614898)	Normal or mild WMH and mild ventriculomegaly	Zivony-Elboum et al. (2012)	
SPG54 (AR)	8p11.23 *DDHD2* (615003)	<2	C	ID or developmental delay, dysarthria, cerebellar signs and short stature (615033) Pathologic lipid peak at 1.3 ppm in brain	TCC, WMH and spinal syrinx	Schuurs-Hoeijmakers et al. (2012)	
SPG55 (AR)	12q24.31 *C12orf65* (613541)	2–7	C	Optic atrophy, muscle atrophy and neuropathy or ID, neuropathy and ophthalmoplegia (615035) Decreased complex I and IV and sometimes V of the respiratory chain	Normal or TCC and WMH	Shimazaki et al. (2012)	Combined oxidative phosphorylation deficiency 7 (Leigh syndrome) (613543)
SPG56^a^ (AR)	4q25 *CYP2U1* ^a^ (615030)			According to OMIM see SPG49^a^			
SPG57 (AR)	3q12.2 *TFG* (602498)	Infancy	C	Optic atrophy and axonal demyelinating motor neuropathy (615658)	Normal	Ishiura et al. (2012) Beetz et al. (2013)	Chondosarcoma extrasqueletal myxoid, fused genes NR4A3/TFG (612237); Motor and sensory neuropathy, AD (604484)
SPG58 (AR, AD?)	17p13.2 *KIF1C* (603060)	2–4	P or C	Mostly complex with ataxia, dysarthria, extrapyramidal chorea, hypotonia, developmental delay or MR and sometimes short stature. Mild phenotype at heterozygous state	Normal or WMH	Dor et al. (2014) Novarino et al. (2014) Caballero Oteyza et al. (2014)	Spastic ataxia SPAX2 (611302)
SPG59 (AR)	15q21.2 *USP8* (603158)	Infancy	C	Nystagmus, pes equinovarus and mild MR	Normal	Novarino et al. (2014)	
SPG60 (AR)	3p22.2 *WDR48* (612167)	Infancy	C	Nystagmus and neuropathy	Normal	Novarino et al. (2014)	
SPG61 (AR)	16p12.3 *ARL6IP1* (603158)	Infancy	C	Motor and sensory polyneuropathy with acropathy mutilation (615685)	Normal or mild dilatation of lateral ventricles	Novarino et al. (2014)	
SPG62 (AR)	10q24.31 *ERLIN1* (611604)	Infancy	P	Pure	Normal	Novarino et al. (2014)	
SPG63 (AR)	1p13.3 *AMPD2* (102771)	Infancy	C	Short stature 615686	WMH, TCC	Novarino et al. (2014)	Pontocerebellar hypoplasia (615809)
SPG64 (AR)	10q24.1 *ENTPD1* (601752)	1–4	C	Amyotrophy, cerebellar signs, moderate ID, aggressiveness, delayed puberty and microcephaly (615683)	WMH	Novarino et al. (2014)	
SPG65 (AR)	10q24.32 q24.33 *NT5C2* (600417)	Infancy	P or C	Amyotrophy, pes equinovarus and learning disability (613162)	TCC, WMH or delayed myelination	Novarino et al. (2014)	
SPG66 (AR)	5q32 *ARSI* (610009)	Infancy	C	Amyotrophy, pes equinovarus and severe sensory/motor polyneuropathy	Corpus callosum and cerebellar hypoplasia, colpocephaly	Novarino et al. (2014)	
SPG67 (AR)	2q33.1 *PGAP1* (611655)	<1–4	C	Amyotrophy	Corpus callosum agenesis, vermis hypoplasia, defective myelination	Novarino et al. (2014)	Complex MR (615802)
SPG68 (AR)	11q13.1 *FLRT1* (604806)	2–3	C	Optic atrophy, nystagmus, mild amyotrophy and peripheral neuropathy	Normal	Novarino et al. (2014)	
SPG69 (AR)	1q31 *RAB3GAP2* (609275)	<1	C	Dysarthria, cataract, deafness and ID	Normal	Novarino et al. (2014)	Martsolf syndrome: (212720); Warburg micro syndrome 2 (614225)
SPG70 (AR)	12q13.3 *MARS* (156560)	<1	C	Amyotrophy and Achilles tendon contracture	ND	Novarino et al. (2014)	Infantile liver failure syndrome (615486); Charcot–Marie–Tooth disease like presentation, AD
SPG71 (AR)	5p13.3 *ZFR* (615635)	Infancy	P	Pure	TCC	Novarino et al. (2014)	
SPG72 (AR/AD)	5q31.2 *REEP2* (609347)	3–4	P	Pure (615625)	ND	Esteves et al. (2014)	
No SPG (AR)	1q21.3 *ADAR1* (146920)	2	P	Pure Increased interferon level	Normal	Crow et al. (2014)	Aicardi–Goutière syndrome (615010); Dyschromatosis symmetrica AD (127400)
No SPG (AR/AD)	9q22.32 *BICD2* (609797)	Infancy	P or C	Pure (AD) or complex with amyotrophy (AR)	Normal	Neveling et al. (2013) Oates et al. (2013) Novarino et al. (2014)	Spinal muscular atrophy AD (615290)
No SPG (AR)	5p15.2 *CCT5* (610150)	Infancy	C	Mutilating sensory neuropathy (256840)	ND	Bouhouche et al. (2006)	
No SPG (AR)	9p13.2 *EXOSC3* (606489)	Infancy	C	Mild cognitive impairment, nystagmus and distal amyotrophy	Cerebellar atrophy or hypoplasia, and enlarged cisterna magna	Wan et al. (2012) Zanni et al. (2013)	Pontocerebellar hypoplasia (614678)
No SPG (AR)	5p15.1 *FAM134B* (613114)	2–3	C	Motor and sensory neuropathy with ulcerations of limbs	Normal	Kurth et al. (2009) Ilgaz-Aydinlar et al. (2014)	Sensory and autonomic neuropathy (HSAN2B) (613115)
No SPG (AR)	1q42.13 IBA57 (615316)	3–12	C	Distal amyotrophy, peripheral neuropathy optic nerve atrophy and reduced visual acuity (SPOAN-like phenotype)	Normal or WMH foci sometimes with TCC and cerebellar atrophy.	Lossos et al. (2015)	Multiple mitochondrial dysfunctions syndrome (615330), Ajit Bolar et al. (2013)
No SPG (AR)	2q24.2 *IFIH1* (606951)	2	P	Pure Increased Interferon level	Normal	Crow et al. (2014)	Aicardi–Goutière syndrome (615846)
No SPG (AR)	1q42.3 *LYST* (606897)	Late (48–58)	C	Cerebellar ataxia, peripheral neuropathy and large peroxidase-positive granules in granulocytes	Mild cerebellar atrophy	Shimazaki et al. (2014)	Chediak–Higashi syndrome (214500)
No SPG (AR)	19q13.1 *MAG* (159460)	Infancy	C	Cerebellar signs, nystagmus, and amyotrophy	Normal	Novarino et al. (2014)	
No SPG (Mito)	*MT*-*ATP6* (516060)	30–50	P or C	Pure or with neuropathy, cerebellar signs and cardiomyopathy	ND	Verny et al. (2011)	Leigh syndrome (551500); Leber optic atrophy (535000); Infantile bilateral striatal necrosis (500003); Epilepsy and lactic acidosis Infantile cardiomyopathy
No SPG (Mito)	*MT*-*CO3* (516050)	Infancy	C	Spastic paraparesis, ophthalmoparesis and lactic acidosis	Basal ganglia hyperintensities (Leigh syndrome-like) and mild cerebral and cerebellar atrophy	Tiranti et al. (2000)	
No SPG (Mito)	*MT*-*TI* (590045)	Adulthood	P or C	Pure with low heteroplasmy levels. Complex with high heteroplasmy levels, with ataxia, deafness, epilepsy, cardiomyopathy and hypogonadism	ND	Corona et al. (2002)	
No SPG (AR)	13q14.3 *RNASEH2B* (610326)	18–21 months	P	Pure	Normal	Crow et al. (2014)	Aicardi–Goutière syndrome (610181)
No SPG (AR)	13q11 *SACS* (604490)	Infancy	C	Spastic ataxia of Charlevoix Saguenay: early childhood onset of cerebellar ataxia, pyramidal tract signs and peripheral neuropathy, ± retinal striations on fundoscopy and thickening of the retinal nerve fiber layer on OCT	Atrophy of the superior cerebellar vermis, hyperintensity of corticospinal tracts	Engert et al. (2000)	
No SPG^a^ (denoted SPG49^a^ by OMIM) (AR)	14q32.31 *TECPR2* ^a^ (615000)	Infancy	C	Severe ID, rigid ataxic gait, brachycephalic microcephaly, fluctuating central hypoventilation, gastroesophageal reflux disease, wake apnea, areflexia and dysmorphic features (615031)	Ventriculomegaly, TCC, cerebral and cerebellar atrophy	Oz-Levi et al. (2012)	
No SPG (AD)	9p13 *VCP* (601023)	54–57	C	Case report: hereditary spastic paraplegia with Paget’s disease of bone.	Normal	Watts et al. (2004) De bot et al. (2012)	Inclusion body myopathy (167320); Amyotrophic lateral sclerosis (613954)

*AD* autosomal dominant, *ALS* amyotrophic lateral sclerosis, *AR* autosomal recessive, *Chr* chromosome, *Cho/Cr and Cho/NAA* ratio choline to creatine or to NAA, *CSF* cerebrospinal fluid, *GM2/3* gangliosides monosialic 2 and 3, *ID* intellectual disability, *LL* lower limb, *Mito* mitochondrial, *MR* mental retardation, *MRI* magnetic resonance imaging, *NAA* *N*-acetyl aspartate, *nb* number, *NBIA* neuronal brain iron accumulation disorders, *ND* not described, *OCT* ocular coherence tomography, *PCr/Pi* ratio of phosphocreatine to inorganic phosphate, *PPM* parts per million, *PMRS* proton magnetic resonance spectrometry, *SPOAN* spastic paraplegia, optic atrophy and neuropathy, *TCC* thin corpus callosum, *UL* upper limb, *WMH* white matter hyperintensity, *y* years

^a^According to the HUGO nomenclature, SPG49 has been associated with *CYP2U1* mutations and SPG56 has not been associated to a specific gene. According to the OMIM numbering, SPG49 has been associated to *TECPR2* mutations and SPG56 to *CYP2U1* mutations

^b^Inheritance mode is indicated when it differs from the one described in families with spasticity

**Fig. 1:**
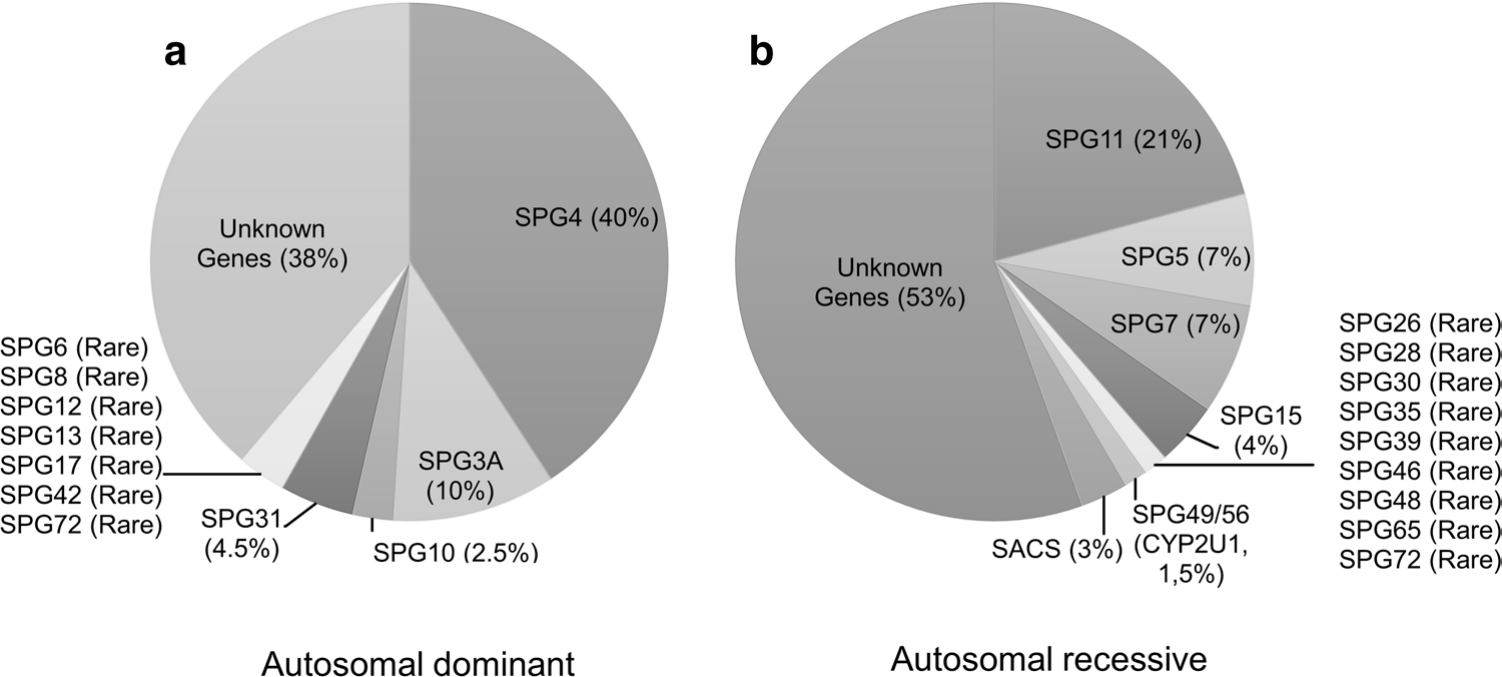
Relative frequencies of the main autosomal dominant (**a**) and recessive (**b**) mutations in the SPATAX (http://spatax.wordpress.com/) cohort (Goizet et al. 2009a, b, 2011; Stevanin et al. 2008a; Tesson et al. (2012); unpublished data)

Autosomal dominant (AD) forms of HSP are mainly pure forms with ages at onset that can range from infancy to late adulthood. Mutations in *SPAST* (SPG4), *ATL1* (SPG3), *KIF5A* (SPG10) and *REEP1* (SPG31) are described as being responsible for around 50 % of all cases (Fig. 1) (Finsterer et al. 2012). *SPAST* point mutations and exonic rearrangements have been implicated in 10–40 % of the HSP patients (Hazan et al. 1999; Meijer et al. 2002; Beetz et al. 2006; Loureiro et al. 2013) and in up to 12 % of sporadic forms (Depienne et al. 2006).

The autosomal recessive (AR) forms appear to be particularly prevalent where consanguinity is common such as in the Middle East or Mediterranean countries (Coutinho et al. 1999; Boukhris et al. 2009; Ruano et al. 2014), and lesser frequent in central Europe, Japan (Takiyama 2014) and USA (with the exception of communities such as the Amish). They are also more complex in clinical terms, associated with greater genetic heterogeneity (Table 2) with an onset of symptoms that is generally early. Only two forms are associated with pure HSP, but this likely results from the assignment of few families each: SPG71 and SPG72. In complex forms, the associated signs may be subtle but important indicators of the mutated gene, such as cerebellar atrophy or cerebellar ataxia with optic atrophy in SPG7, developmental delay and short stature in SPG20 (Troyer syndrome), dysarthria, distal amyotrophy, premature aging and cognitive decline in SPG21 (Mast syndrome), peripheral neuropathy and abnormal skin and hair pigmentation in SPG23 (Lison syndrome) (Table 2). Mental retardation or intellectual deterioration, thin corpus callosum (TCC) and axonal neuropathy are highly suggestive of SPG11 (Stevanin et al. 2008a). Finally, spastic ataxia with dysarthria, nystagmus and retinal striations is suggestive of autosomal recessive spastic ataxia of Charlevoix-Saguenay (ARSACS). Mutations in the *CYP7B1* (SPG5), *SPG7*, *KIAA1840* (SPG11) and *ZFYVE26* (SPG15) genes are among the most frequently found but their relative frequencies vary according to the geographical origin (Stevanin et al. 2008a; Paisan-Ruiz et al. 2008; Erichsen et al. 2009; Goizet et al. 2009a; Schüle et al. 2009b; Arnoldi et al. 2012; Klebe et al. 2012a; Pfeffer et al. 2014) (Fig. 1). Point mutations or rearrangements in *KIAA1840* (SPG11) have been shown to account for approximately 20 % of AR-HSP (Stevanin et al. 2008a).

X-linked forms are rare and include two clinical entities well recognized by pediatricians (Table 2): SPG1, caused by mutations in the neural cell adhesion molecule *L1CAM* gene, and SPG2, which results from mutations in the gene encoding the proteolipid protein (*PLP1*), a myelin component. SPG2 can also account for late-onset cases in women (Sivakumar et al. 1999).

## Phenotype–genotype correlations in HSP

Many studies have failed in the past to determine reliable phenotype–genotype correlations. However, the systematic analysis of a large set of genes, including exome sequencing, is regularly unmasking unusual phenotypes and inheritance modes associated with mutations in HSP genes and the nature of the mutation in some of them can now be correlated to a specific phenotype.

### Instances where similar mutations are associated with a wide spectrum of HSP phenotypes; extension of the clinical picture previously observed

There are good examples of variable phenotypes among HSP subtypes, as SPG4 in which age at onset can vary from early childhood to asymptomatic status at old ages. As more families are reported with a mutation in a specific gene, the full spectrum of each genetic entity extends and there are now fewer than ten HSP loci/genes associated exclusively with pure forms of the disease, most of them accounting for single or only a few families so far (Table 2; Supplementary Table 1). This was the case, for example, with SPG10, which was initially thought to be a pure form but now also accounts for 10 % of the complex AD families (Goizet et al. 2009b). In SPG7, the occurrence of cerebellar ataxia and/or atrophy (Klebe et al. 2012a) or progressive external ophthalmoplegia (Wedding et al. 2014; Pfeffer et al. 2014) suggests that the analysis of this gene should be extended to other phenotypes. Patients with isolated optic neuropathy should also be tested for mutations on *SPG7* (Klebe et al. 2012a).

### Instances where the nature of the mutations of a specific HSP gene can determine the inheritance model and/or associated phenotype

One of the recent advances in HSP genetics is the identification of various modes of inheritance of the mutations in single HSP genes. This is what occurs for *REEP2* mutations that have recently been implicated in three families with recessive or dominant transmission of a pure HSP, namely SPG72 (Novarino et al. 2014; Esteves et al. 2014). In one Portuguese family with AR inheritance, two mutations segregated in trans including a splice site mutation leading to a loss of function of the corresponding allele and a missense mutation responsible for reduced binding capacities to membranes of the protein formed from the second allele. In a French autosomal dominant family, the disease segregated with a heterozygous missense mutation that had a dominant negative effect on the capacity of the wild-type protein to bind membranes. In both cases, AD and AR mutations led to a complete loss of membrane binding capacities of the REEP2 protein with consequences for the tubular structure of the endoplasmic reticulum (ER) (Esteves et al. 2014). Recently, position p.Arg415 of Atlastin-1 (SPG3A) was shown to be a hotspot for missense mutations, first associated with incomplete penetrance with an AD inheritance pattern (D’Amico et al. 2004), and then with AR transmission (Varga et al. 2013) (Khan et al. 2014). Similarly, an unusual recessive or dominant inheritance has been suggested in *SPAST* (SPG4, Lindsey et al. 2000) and *SPG7* (McDermott et al. 2001; Sánchez-Ferrero et al. 2013), respectively.

The nature of the mutation and/or its localization in the protein can sometimes impact both the inheritance model and the phenotype at the same time, so that the nature of the mutation can predict the phenotype. This was observed with *KIF1A* (SPG30), in which missense homozygous mutations located in the kinesin motor domain account for a relatively pure HSP (Erlich et al. 2011; Klebe et al. 2012b), whereas heterozygous mutations located in the ATP binding site of KIF1A were found in patients with severe mental retardation with axial hypotonia, peripheral spasticity and mild atrophy of cerebellum and or corpus callosum, a phenotype reminiscent of SPG11 (Hamdan et al. 2011; Chang et al. 2014). Homozygous *KIF1A* frameshift mutations lead to hereditary sensory neuropathy type IIC (Rivière et al. 2011). Similarly, heterozygous mutations in *HSPD1* lead to SPG13 (Hansen et al. 2002), but homozygous missense mutations of the same gene are implicated in hypomyelinating leukodystrophy type 4 (Magen et al. 2008). Mutations in *TFG* are responsible for SPG57, an AR-HSP associated with optic atrophy and neuropathy (Beetz et al. 2013) but can also be responsible for AD motor and sensory neuropathy (Ishiura et al. 2012). Interestingly, the *TFG* mutations affect different domains of the protein: the coil–coil domain in the HSP family, the P/Q rich domain in the family with neuropathy, suggesting different pathological mechanisms. In addition, one patient with neuropathy had ubiquitin- and TDP43-positive cytoplasmic neuronal inclusions reminiscent of amyotrophic lateral sclerosis (ALS) (Supplementary Table 2), suggesting a toxic gain of function effect resulting in a dominant inheritance pattern. In contrast, biallelic mutations affect the capacity of TFG to self-assemble and then probably lead to a loss of function (Beetz et al. 2013). This is also the case with *REEP1*, in which frameshift mutations or missense mutations that abolish ER targeting and affect the capacity of the protein to bind ATL1 (Falk et al. 2002; Beetz et al. 2012) lead to HSP (Züchner et al. 2006; Beetz et al. 2008; Hewamadduma et al. 2009; Goizet et al. 2011), whereas in-frame deletions do not impact the capacity of the protein to bind ATL1 and lead to hereditary motor neuropathy type V (Beetz et al. 2012).

### Instances where the mutations of a specific HSP gene lead to overlapping diseases

During the past few years, it has appeared that HSP and other neurological conditions are at opposite ends of a continuum of overlapping diseases. The clinical overlap of HSP with peripheral neuropathies, cerebellar ataxias or mental disabilities is not new since mutated HSP patients can have a clinical presentation associating symptoms specific to these groups of disorders. ARSACS is a good illustration of this clinical overlap between ataxias and spastic paraplegias (Bouhlal et al. 2011). It is sometimes difficult to decide which symptom is most prominent in the clinical presentation and this may also depend on the physician’s expertise. The overlap between HSP and ataxias was again recently highlighted by mutations in *GBA2* (SPG46), which have been found in patients with spastic ataxia associated with cataract, having ataxia (Hammer et al. 2013; Votsi et al. 2014) or spasticity (Martin et al. 2013; Citterio et al. 2014) as the prominent clinical feature. Point mutations and exonic deletions in *KIF1C* have both been reported in HSP (Novarino et al. 2014) and spastic ataxia (Dor et al. 2014), illustrating the fact that both diseases are part of the same clinical spectrum. *PNPLA6* mutations are found in patients with a wide range of phenotypes: Gordon Holmes spinocerebellar syndrome (ataxia with brisk reflexes and hypogonadism), Boucher-Neuhäuser syndrome (ataxia with chorioretinal dystrophy and hypogonadism), isolated cerebellar ataxia and isolated spastic paraplegia (SPG39) (Rainier et al. 2008; Synofzik et al. 2014).

**Fig. 2:**
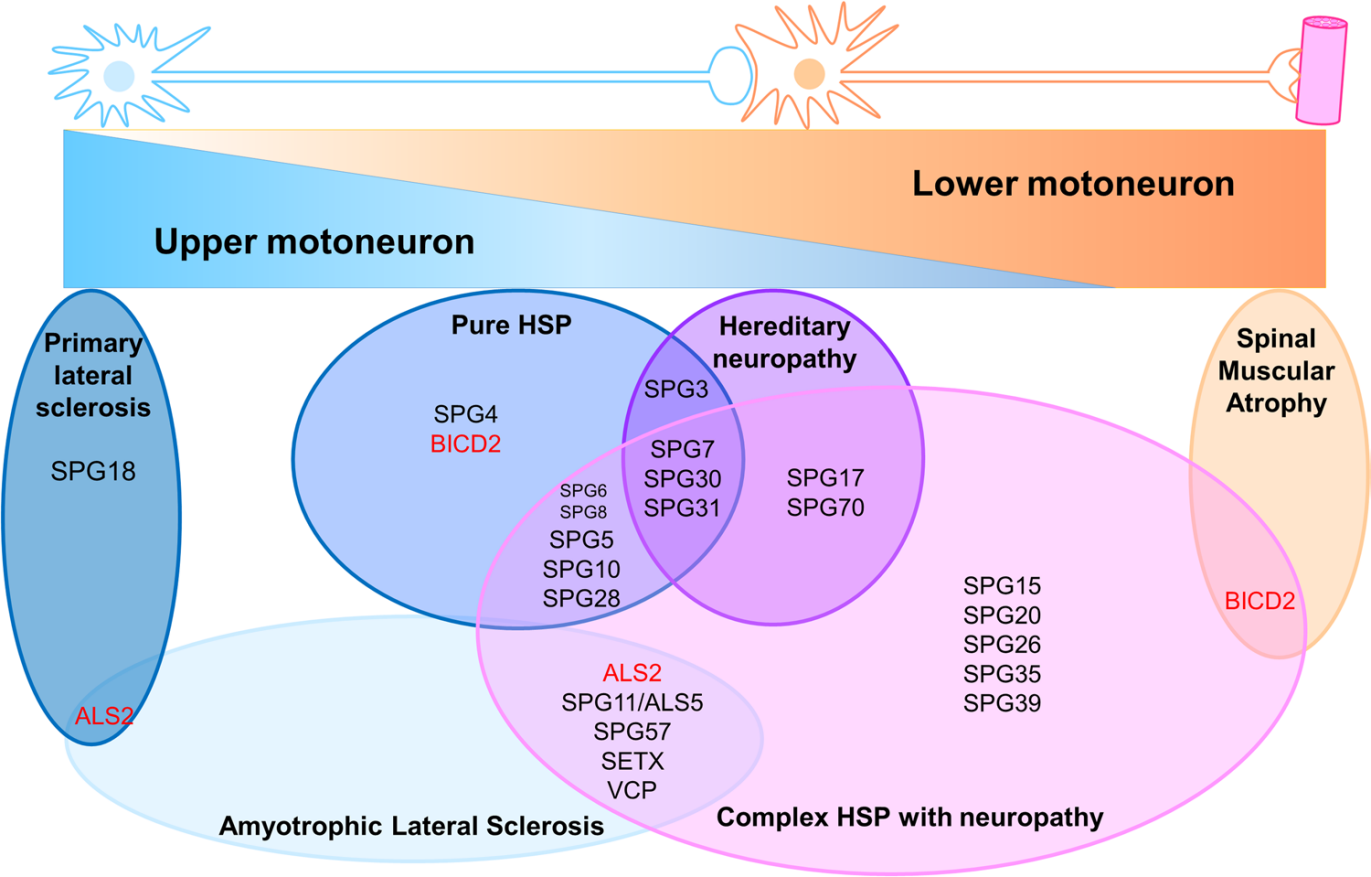
Clinico-genetic entities associated with hereditary spastic paraplegia (HSP) according to the motor neuron phenotypic presentation. When mutated, HSP genes can be associated with various phenotypes that overlap with upper and lower motor neuron diseases

Regarding motor neuron diseases and polyneuropathies (Fig. 2), HSP genes have been found mutated in patients with (i) peripheral nerve affections such as Charcot–Marie–Tooth (CMT) neuropathies, (ii) first and secondary motor neuron degeneration, such as ALS, and (iii) lower motor neuron disorders such as spinal muscular atrophy (SMA). For example, *KIAA1840* *(SPG11)* mutations may mimic ALS5 when muscle wasting is marked in absence of other complicated signs, or complex HSP in presence of cerebellar and cognitive signs (Stevanin et al. 2007b, 2008a; Orlacchio et al. 2010; Daoud et al. 2012; Romagnolo et al. 2014). Similarly, mutations in *TFG* can also be associated with HSP (SPG57) and ALS-like presentations (Ishiura et al. 2012; Beetz et al. 2013). *BIDC2* mutations are mainly responsible for SMA phenotypes but can cause HSP as well (Neveling et al. 2013; Oates et al. 2013). *ERLIN2* mutations are responsible for SPG18 (Yıldırım et al. 2011; Alazami et al. 2011; Wakil et al. 2013) but also account for juvenile primary lateral sclerosis, another neurodegenerative disorder of the upper motor neuron overlapping HSP phenotype (Al-Saif et al. 2012). Finally, missense mutations in *KIF5A* affecting the kinesin motor domain, or in *MARS* (SPG70) encoding the methionyl-tRNA synthase essential for protein biosynthesis (Deniziak and Barciszewski 2001), are responsible for pure (Reid et al. 2000) or complex forms of HSP with neuropathy or amyotrophy (Tessa et al. 2008; Goizet et al. 2009b; Musumeci et al. 2011; Crimella et al. 2012; Collongues et al. 2013; Novarino et al. 2014; Liu et al. 2014) but also CMT (Crimella et al. 2012; Gonzalez et al. 2013; Liu et al. 2014). In *MARS*, the mutations identified in CMT-like disease and in HSP patients are located in different domains.

Other examples are the *FA2H* and *C9ORF12* genes, which can be found mutated in patients with neuronal brain iron accumulation (NBIA) or spastic paraplegia (Edvardson et al. 2008; Schneider and Bhatia 2010; Dick et al. 2010; Kruer et al. 2010; Hartig et al. 2011; Landouré et al. 2013). There are also two genes in which mutations are associated with complex HSP but also account for pontocerebellar hypoplasia: *AMPD2* (SPG63) and *EXOSC3* (Wan et al. 2012; Zanni et al. 2013; Novarino et al. 2014). *GJC2* accounts for complex HSP associated with dysarthria, cerebellar ataxia and mental impairment (Orthmann-Murphy et al. 2009) and hypomyelinating leukodystrophy Pelizaeus–Merzbacher-like disease (Uhlenberg et al. 2004). Similarly, *PGAP1* and *C12ORF65* have been reported mutated in encephalomyopathies (Antonicka et al. 2010; Murakami et al. 2014), but they are also mutated in patients with complex HSP (Shimazaki et al. 2012; Tucci et al. 2013; Spiegel et al. 2014; Novarino et al. 2014).

### Instances where mutations can either account for HSP or multisystemic disorders

Several genes mutated in HSP can also be responsible for multisystemic disorders in which spasticity can be part of the phenotype or even absent. Some of these genes cause developmental disorders such as those involving mutations in genes coding for adaptor protein complex 4 (AP4) (SPG47, 50, 51 and 52) (Moreno-De-Luca et al. 2011; Abou Jamra et al. 2011). *KIAA0196* is mutated in pure AD-HSP (SPG8) patients (Valdmanis et al. 2007), but also in complex cases in which the spasticity decreased upon levodopa treatment (Bettencourt et al. 2013), and recently a homozygous splice site mutation leading to exon 27 skipping was involved in Ritscher–Schinzel syndrome, a developmental syndrome with craniofacial abnormalities, congenital heart defects and cerebellar brain malformations (Elliott et al. 2013).

Biallelic mutations in *IBA57*, encoding a Fe/S mitochondrial protein assembly factor, can be responsible for a slowly progressive childhood-onset HSP (Lossos et al. 2015), up to multiple mitochondrial dysfunction syndrome 3 (MMDS3; OMIM #615330), a severe lethal encephalopathy with multiple malformations, myopathy and hyperglycinemia (Ajit Bolar et al. 2013). Mutations in *LYST* were first described in Chediak–Higashi syndrome, a disease characterized by decreased pigmentation, photophobia, nystagmus and abnormal susceptibility to infection (Barbosa et al. 1997). Recently, one mutation was reported in a family with late-onset complex HSP with cerebellar ataxia, peripheral neuropathy and large peroxidase-positive granules in granulocytes (Shimazaki et al. 2014).

Finally, biallelic mutations in *BSCL2* are responsible for congenital generalized lipodystrophy type 2, characterized by severe lipoatrophy, insulin resistance, hypertriglyceridemia and mental retardation (Magré et al. 2001). In contrast, heterozygous missense mutations in its *N*-glycosylation motif (p.Asn88Ser and p.Ser90Leu) result in a toxic gain of function by ER-stress-mediated cell death responsible for motor neuron diseases, including SPG17 and hereditary motor neuropathy type V (Windpassinger et al. 2004; Ito and Suzuki 2009).

### Modifying factors

One striking observation is the large interfamilial but also intrafamilial phenotypic variability regarding age at onset and disease severity between patients, particularly in autosomal dominant HSP subtypes. Some SPG4 patients within the same family and carrying the same mutation may indeed remain asymptomatic throughout their life whereas others have an early onset and are severely affected and may present with additional features such as cognitive impairment and peripheral neuropathy. One major issue is therefore the identification of environmental or genetic modifiers in HSP. This has been especially studied in *SPAST*-positive patients; three modifier variants have been suggested so far: the rare p.Ser44Leu and p.Pro45Gln polymorphisms in *SPAST* and the p.Gly563Ala polymorphism located in *HSPD1* (HSP60/SPG13) (Svenson et al. 2004; Bross et al. 2008). Large population studies are still lacking to validate these findings.

Phenotypic variability is well known in mitochondrial disorders. The mitochondrial *MT*-*TI* mutation segregating in a HSP family is associated with a disease severity that is correlated to the level of heteroplasmy: from pure HSP in the proband showing 55 % heteroplasmy levels in muscles, to a multisystemic disorder with cardiomyopathy in the brother with heteroplasmy levels of 90 % (Corona et al. 2002).

Sex-dependent penetrance or severity has been suggested for *SPAST* mutations on the basis of a significant excess of affected males (Starling et al. 2002; Proukakis et al. 2011) and/or an earlier age at onset (Mitne-Neto et al. 2007) This was also suggested for *ATL1* mutations (Varga et al. 2013; Luo et al. 2014).

In other cases, a variant may be pathogenic when inherited as a recessive trait but may become a susceptibility factor for other neurological conditions at the heterozygous state. Recent observations in the gene encoding paraplegin are striking. SPG7 patients usually have pure or complex forms of HSP, mainly associated with cerebellar signs and optic atrophy. Interestingly, heterozygous carriers of *SPG7* truncating mutations can develop a late-onset cerebellar syndrome/atrophy without spasticity, suggesting a predisposition for late-onset neurodegenerative disorders of heterozygous SPG7 carriers, mimicking an autosomal dominant inheritance when children are carrying two causative mutations. Moreover, patients with the heterozygous p.Asp411Ala mutation were reported with optic atrophy without ataxia or spasticity in a large autosomal dominant kindred. *SPG7* has, therefore, to be considered in patients with late-onset cerebellar signs or optic atrophy, even in the absence of spasticity (Klebe et al. 2012a).

## Genetic diagnosis of HSP

A genetic diagnosis workflow to be used in routine diagnosis is proposed in Supplementary Fig. 1 when new technologies are still not available. In sporadic and AD cases, *ATL1* and *SPAST* genes should be tested first depending on age at onset. Of note, up to 12 % of sporadic cases are mutated in the *SPAST* gene (Depienne et al. 2006; Beetz et al. 2006) while up to 40 % are mutated among autosomal dominant forms. Since 50 % of the mutations in SPG4 are larger deletions, duplications or complex genomic rearrangements (Beetz et al. 2006; Depienne et al. 2007), a dedicated technique (array-CGH, MLPA) has to be used in parallel. In AR cases, the genes to be tested are based on the associated phenotype. Indeed, the relative frequency of SPG11 varies according to phenotype (Stevanin et al. 2008a). SPG11 accounts for <1 % of patients with a pure phenotype, 4.5 % of cases with spastic paraplegia and cognitive impairment without thinning of the corpus callosum (TCC), but up to 59 % of persons with early-onset progressive spasticity with mild intellectual disability and/or cognitive decline associated with TCC. The other HSP genes are analyzed depending on the inheritance mode, the clinical presentation and the results of additional examinations.

However, the number of causative genes is growing very rapidly thanks to the improvement of sequencing techniques, and the classical testing of one gene after the other is progressively being replaced by diagnostic kits allowing multiple genes to be tested in parallel. Access to all SPG variants with such techniques may open opportunities to analyze their modifier effects in addition to the causative mutation in the near future. This may also represent a challenge for the interpretation of their effects since multiple variants with potential effects will likely be identified in more than one candidate gene. The development of common databases and of bioinformatics but also biological pipelines for analysis of the pathogenic effects will be required and will sometimes complicate the diagnosis workflow.

When a mutation is identified, genetic counseling and prenatal and presymptomatic testing are possible options.

## Physiopathology of HSP

Known HSP genes encode proteins involved mainly in ER morphogenesis, microtubule dynamics and transport, mitochondrial quality control, lipid metabolism and endosomal/lysosomal functions (Table 3), and collectively suggest that HSP might be caused by impaired cellular trafficking (Stevanin et al. 2008b; Blackstone 2012).

**Table 3:** Functions of the proteins encoded by the genes involved in hereditary spastic paraplegia

SPG no (HUGO nomenclature)	*Gene* (*OMIM no*)	Chromosome	Protein	Protein function
SPG1	*L1CAM* (308840)	Xq28	L1 cell adhesion molecule	Axonal guidance
SPG2	*PLP1* (300401)	Xq22.2	Proteolipid protein 1	Myelin component Oligodendrocyte progenitor cell migration
SPG3 SPG3A	*ATL1* (606439)	14q22.1	Atlastin GTPase 1	Formation of the tubular ER Dendritic morphogenesis Inhibit BMP signaling
SPG4	*SPAST* (604277)	2p22.3	Spastin	Microtubule dynamics, BMP signaling
SPG5/SPG5A	*CYP7B1* (603711)	8q12.3	Cytochrome P450, family 7, subfamily B, polypeptide 1	Hydroxylase, cholesterol and neurosteroïd metabolism
SPG6	*NIPA1* (608145)	15q11.2	NIPA1/non-imprinted in Prader Willi/Angelman syndrome 1	Mg^2+^ transporter Inhibitor of BMP pathway
SPG7	*SPG7* (602783)	16q24.3	Paraplegin	Component of the m-AAA protease
SPG8	*KIAA0196* (610657)	8q24.13	Strumpellin	Actin remodeling
SPG10	*KIF5A* (602821)	12q13.3	Kinesin heavy chain isoform 5A	Motor protein, axonal transport
SPG11	*KIAA1840* (610844)	15q21.1	Spatacsin	Lysosome shaping
SPG12	*RTN2* (603183)	19q13.32	Reticulon 2	ER shaping
SPG13	*HSPD1* (118190)	2q33.1	Heat shock 60 kDa protein 1/chaperonin	Mitochondrial chaperone
SPG15	*ZFYVE26* (612012)	14q24.1	Spastizin	Lysosome shaping, cytokinesis, autophagy
SPG17	*BSCL2* (606158)	11q12.3	Seipin	ER protein, scaffolding protein for lipid metabolism and lipid droplet formation
SPG18	*ERLIN2* (611605)	8p11.23	SPFH2	ER-associated degradation pathway (ERAD)
SPG20	*KIAA0610* (607111)	13q12.3	Spartin	Cytokinesis, BMP signaling, Lipid droplet maintenance, Mitochondrial Ca^2+^ homeostasis
SPG21	*ACP33* (608181)	15q22.31	Maspardin	Associated predominantly with markers for the trans-Golgi and endocytic compartments
SPG22	*SLC16A2* (300095)	Xq13.2	Solute carrier family 16 (monocarboxylic acid transporter) member 2	Thyroid hormone transporter
SPG26	*B4GALNT1* (601873)	12q13.3	Beta-1,4-*N*-acetyl-galactosaminyl transferase 1	Ganglioside metabolism
SPG28	*DDHD1* (614603)	14q22.1	DDHD domain containing 1	Phospholipase A1, lipid metabolism
SPG30	*KIF1A* (601255)	2q37.3	Kinesin family member 1A	Motor protein, axonal anterograde transport
SPG31	*REEP1* (609139)	2p11.2	Receptor expression-enhancing protein 1	ER-shaping, mitochondrial functions?
SPG33	*ZFYVE27* (610244)	10q24.2	ZFYVE27/Zinc Finger, FYVE domain containing 27/Protrudin	ER morphology Neurite outgrowth
SPG35	*FA2H* (611026)	16q23.1	Fatty acid 2-hydroxylase	Myelin stability Cell differentiation
SPG39	*PNPLA6* (603197)	19p13.2	Patatin-like phospholipase domain containing 6/neuropathy target esterase (NTE)	Lipid metabolism Membrane curvature
SPG42	*SLC33A1* (603690)	3q25.31	Solute carrier family 33 Acetyl-CoA transporter, member 1	Acetyl-CoA transporter
SPG43	*C19orf12* (614297)	19p13.11-q12	Chromosome 19 open reading frame 12	–
SPG44	*GJC2* (608803)	1q42.13	Gap junction protein, gamma 2, 47 kDa	Oligodendrocyte connexin
SPG46	*GBA2* (609471)	9p13.3	Glucocerebrosidase 2	Lipid metabolism
SPG47	*AP4B1* (607245)	1p13.2	Adaptor-related protein complex 4, beta 1 subunit	Membrane trafficking
SPG48	*AP5Z1* (613653)	7p22.1	Adaptor-related protein complex 5, zeta 1 subunit	Membrane trafficking
SPG49^a^ (denoted SPG56^a^ by OMIM)	*CYP2U1* ^a^ (615030)	4q25	Cytochrome P450, family 2, subfamily U, polypeptide 1	Lipid metabolism
SPG50	*AP4M1* (602292)	7q22.1	Adaptor-related protein complex 4, mu 1 subunit	Membrane trafficking
SPG51	*AP4E1* (607244)	15q21.2	Adaptor-related protein complex 4, epsilon 1 subunit	Membrane trafficking
SPG52	*AP4S1* (607243)	14q12	Adaptor-related protein complex 4, sigma 1 subunit	Membrane trafficking
SPG53	*VPS37A* (609927)	8p22	Vacuolar protein sorting 37 homolog A	Member of the ESCRT-I complex
SPG54	*DDHD2* (615003)	8p11.23	DDHD domain containing 2	Phospholipase, lipid metabolism
SPG55	*C12orf65* (613541)	12q24.31	Chromosome 12 open reading frame 65	Member of the mediated ribosome rescue system in mitochondria
SPG56^a^	*CYP2U1* ^a^ (615030)	4q25	See SPG49^a^ and *TECPR2* ^a^	
SPG57	*TFG* (602498)	3q12.2	TRK-fused gene	ER morphology, vesicle transport between ER and Golgi
SPG58	*KIF1C* (603060)	17p13.2	Kinesin family member 1C	Motor protein, retrograde Golgi to ER transport
SPG59	*USP8* (603158)	15q21.2	Ubiquitin specific peptidase 8	Deubiquitination enzyme
SPG60	*WDR48* (612167)	3p22.2	WD repeat domain 48	Deubiquitination regulation
SPG61	*ARL6IP1* (603158)	16p12.3	ADP-ribosylation factor-like 6 interacting protein 1	ER morphology
SPG62	*ERLIN1* (611604)	10q24.31	ER lipid raft associated 1	ER-associated degradation
SPG63	*AMPD2* (102771)	1p13.3	Adenosine monophosphate deaminase 2	Deaminates AMP to IMP in purine nucleotide metabolism
SPG64	*ENTPD1* (601752)	10q24.1	Ectonucleosidase triphosphate diphosphorylase 1	Hydrolyzes ATP and other nucleotides to regulate purinergic transmission
SPG65	*NT5C2* (600417)	10q24.32 q24.33	Cytosolic 5′-nucleotidase	Hydrolyses IMP in both purine/pyrimidine nucleotide metabolism
SPG66	*ARSI* (610009)	5q32	Arysulfatase I	Hydrolyses sulfate esters, hormone biosynthesis
SPG67	*PGAP1* (611655)	2q33.1	GPI inositol deacylase	GPI-AP sorting by ERES
SPG68	*FLRT1* (604806)	11q13.1	Fibronectin leucine rich transmembrane protein 1	FGF pathway
SPG69	*RAB3GAP2* (609275)	1q31	RAB3 GTPase activating protein subunit 2	ER morphology
SPG70	*MARS* (156560)	12q13.3	Methionyl-tRNA synthetase	Cytosolic methionyl-tRNA synthetase
SPG71	*ZFR* (615635)	5p13.3	Zinc finger RNA binding protein	–
SPG72	*REEP2* (609347)	5q31.2	Receptor expression-enhancing protein 2	ER shaping
No SPG	*ADAR1* (146920)	1q21.3	Adenosine deaminase RNA-specific	RNA metabolism
No SPG	*BICD2* (609797)	9q22.32	Bicaudal D homologue 2	Adaptor protein of the dynein–dynactin motor complex
No SPG	*CCT5* (610150)	5p15.2	Chaperonin containing TCP1, subunit 5	Cytosolic chaperonin
No SPG	*EXOSC3* (606489)	9p13.2	Exosome component 3	Core component of the RNA exosome complex
No SPG	*FAM134B* (613114)	5p15.1	FAM134B	Golgi protein
No SPG	*IFIH1* (606951)	2q24.2	Interferon-induced helicase C domain containing protein 1	Interferon signaling
No SPG	*LYST* (606897)	1q42.3	Lysosomal trafficking regulator protein	Lysosome fusion/fission regulation
No SPG	*MAG* (159460)	19q13.1	Myelin-associated glycoprotein	Myelination
No SPG	*MT*-*ATP6* (516060)	*Mitochondrial*	Complex V, ATP synthase, subunit ATPase 6	Respiratory chain complex V subunit
No SPG	*MT*-*CO3* (516050)	*Mitochondrial*	Cytochrome c oxydase III/Complex IV	Respiratory chain complex IV subunit
No SPG	*MT*-*TI* (590045)	*Mitochondrial*	Isoleucine transfer RNA (Mitochondrial)	Mitochondria
No SPG	*RNASEH2B* (610326)	13q14.3	Ribonuclease H2 subunit B	Metabolism of ribonucleotides
No SPG	*SACS* (604490)	13q11	Sacsin	Chaperone
No SPG (denoted SPG49^a^ by OMIM)	*TECPR2* ^a^ (615000)	14q32.31	Tectonin beta-propeller repeat containing 2	Autophagy pathway
No SPG	*VCP* (601023)	9p13	Valosin-containing protein	Member of the AAA+ family; Role in the ubiquitin-proteasome system
No SPG	*IBA57* (615316)	1q42	Iron–sulfur cluster assembly homolog	Part of the iron–sulfur cluster (ISC) assembly machinery in mitochondria

*(m)AAA* (mitochondrial) ATPase associated with diverse cellular activities, *BMP* bone morphogenetic pathway, *ER* endoplasmic reticulum, *ERES* ER exit sites, *ESCRT* endosomal sorting complexes required for transport, *FGF* fibroblast growth factor, *GPI-AP* glycosylphosphatidylinositol-anchor protein, *IMP* inositol monophosphate

^a^According to the HUGO nomenclature, SPG49 has been associated with *CYP2U1* mutations and SPG56 has not been associated to a specific gene. According to the OMIM numbering, SPG49 has been associated to *TECPR2* mutations and SPG56 to *CYP2U1* mutations

Clear evidence of impaired trafficking comes from the involvement of KIF5A (SPG10), KIF1A (SPG30) and KIF1C (SAX2/SPAX2/SPG58), which are motor proteins (kinesins) involved in organelle/vesicle trafficking. In KIF5A, heterozygous missense mutations in the motor domain are associated with a reduced velocity along microtubules in gliding assays (Ebbing et al. 2008). There is also other evidence of trafficking disturbances in HSP. Neurons from knockout (KO) mice for *Spast* present with a marked impairment of microtubule dynamics along axons, accompanied by axonal swelling and cargo stalling (Tarrade et al. 2006; Kasher et al. 2009; Fassier et al. 2013). Spastin (SPAST/SPG4) is a microtubule-severing protein, which links cytoskeletal dynamics to membrane remodeling in several cellular processes. Abnormal axonal swellings have been also reported in *Plp1*, *Fa2h* and *Kif5a* KO mice and/or fly models (Edgar et al. 2004; Potter et al. 2011; Füger et al. 2012; Karle et al. 2012). Axon swellings with accumulation of membranous material in axons have also been observed in *Spg7* KO mice (Ferreirinha et al. 2004) and in nerve biopsies of SPG11 patients (Hehr et al. 2007). Finally, axonal trafficking of vesicles was shown to be impaired in neurons derived from induced pluripotent stem cells (IPSC) of SPG11 patients (Pérez-Brangulí et al. 2014).

The best known example of mitochondrial dysfunction in HSP is related to the SPG7 subtype. The corresponding gene is the first to have been identified in HSPs, in 1998 (Casari et al. 1998) and encodes paraplegin. Paraplegin is a conserved subunit of the ATP-dependent m-AAA protease of the inner membrane of the mitochondria involved in the quality control of multiple proteins of the respiratory pathway. SPG7 is associated with multiple mitochondrial DNA deletions, suggesting that functions of other mitochondrial proteins involved in either mitochondrial DNA replication itself or pathways of mitochondrial quality control are altered (Wedding et al. 2014). As a consequence, SPG7 patients present with reduced mitochondrial respiration rates and increased sensitivity to oxidative stress (Atorino et al. 2003). *Spg7* KO mice show axonal swellings with accumulation of membranous material and mitochondria in distal axons (Ferreirinha et al. 2004) reminiscent of what is observed in *Spg4/Spast* KO mice and therefore making the link between mitochondrial alterations and intracellular trafficking defects. Recently, reduced levels and activities of mitochondrial 4Fe-4S mitochondrial proteins have been observed in a family with a combination of spastic paraplegia, optic atrophy, and peripheral neuropathy (SPOAN) due to *IBA57* mutations (Lossos et al. 2015). Finally, impaired mitochondrial motility was shown in neurons derived from iPSC of SPG3A patients (Zhu et al. 2014), again linking trafficking and mitochondrial functions; mitochondrial distribution is dependent on microtubule cytoskeleton and tubular ER functions.

The number of HSP proteins involved in the functions of the ER is growing. Six genes related to these functions are mutated in HSP (Goyal and Blackstone 2013). Atlastin-1 is a GTPase able to promote ER tubule homotypic fusion by forming trans-oligomeric complexes between two adjacent ER tubules (Orso et al. 2009). Rismanchi et al. (2008) showed ER morphology effects of the mutant atlastin-1 while ER-Golgi trafficking was largely unaffected. Another subgroup of proteins acts in ER shaping: ARL6IP1 (SPG61), reticulon 2 (SPG12), REEP1 (SPG31), REEP2 (SPG72) and RAB3GAP2 (Montenegro et al. 2012; Novarino et al. 2014; Esteves et al. 2014). Of note, spastin, atlastin-1 and REEP1 have been found to interact with each other and to act on microtubule interactions with the tubules of the ER (Park et al. 2010).

The secretory pathway is also altered. Spatacsin (SPG11) and spastizin (SPG15) account for proteins involved in the formation of lysosomes (Chang et al. 2014) and interact with components of the AP5 complex involved in membrane sorting of late endosomes (Hirst et al. 2013). Another adaptor protein complex, AP4, is also involved in neurodevelopmental diseases overlapping with HSP: SPG50, SPG51, SPG47 and SPG52 (Moreno-De-Luca et al. 2011; Abou Jamra et al. 2011). Accumulation of giant lysosomes and autophagosomes was observed in patient’s cells and in a Drosophila KO for *Lyst*, suggesting that LYST plays a role in homotypic fusion of these organelles (Rahman et al. 2012). BICD2 is an adaptor protein necessary for retrograde transport of vesicles from ER to Golgi (Heffernan and Simpson 2014). Finally, the NIPA1 protein (SPG6) is a neuron-specific transmembrane protein principally localized in the early endosomal compartment and on the plasma membrane, and its ortholog in Drosophila (Spict) was shown to interact with bone morphogenetic protein (BMP) receptors and promote their internalization from the membrane (Wang et al. 2007). BMP signaling is necessary for normal microtubule cytoskeleton assembly, and *NIPA1* mutants are less efficient in the lysosomal degradation of BMP receptors, therefore, interfering with distal axonal functions (Tsang et al. 2009).

There are some subtypes of HSP that affect multiple brain regions and are associated with an early onset of the symptoms, which include psychomotor delay. These disorders include the SPGs affecting the AP4 complex as well as SPG1 and SPG2. In SPG3A and SPG11, the early onset of the disease may also suggest an abnormal development but information is lacking to confirm this point. A good example of abnormal development is SPG1. L1CAM function is necessary for correct formation of the corticospinal tract. Indeed, mice lacking *L1cam* mimic the human phenotype and present with defects in axonal guidance in the corticospinal tracts and reduced decussation (Cohen et al. 1998). Of note, abnormal development leading to psychomotor delay has also been suspected in HSP due to mutations in *PGAP1* (SPG67) (Novarino et al. 2014).

Some genes expressed almost exclusively in glial cells have been identified in HSP, such as *SLC16A2* coding for MCT8, a thyroid hormone transporter expressed by astrocytes during embryonic development in mammals. Several other proteins involved in HSP are expressed predominantly in non-neuronal cells, such as MAG and PLP1, two components of myelin, and FA2H, an enzyme involved in the hydroxylation of sphingolipids, galactolipids and other fatty acids (Hiroko 2010). Mice lacking *Fa2h* show a degeneration of myelin sheaths at 18 months (Zöller et al. 2008). PLP1/DM20 is expressed in oligodendrocytes and oligodendrocyte progenitor cells (OPC). Despite normal myelination, mice lacking *Plp1* have physically fragile myelin and a decrease of its cholesterol content (Werner et al. 2013). It can be suggested that PLP1/DM20 may stabilize and maintain the myelin sheath. Moreover, these mice have an alteration of fast retrograde and anterograde transport (Edgar et al. 2004).

Lipid metabolism is an emerging pathway in HSP, but its importance is growing daily and has opened an entirely novel perspective on the pathogenesis of this group of diseases. There is increasing evidence that lipids have critical roles as signaling mediators and effectors, and that lipid composition of neuronal membranes affects crucial processes such as exocytosis and ion channel functions, and contributes to the formation of membrane domains. As an example, the loss of the B4GALNT1, an enzyme of the catabolism of complex gangliosides, changes the cholesterol and phospholipid content of membranes (Ohmi et al. 2011). *B4galnt1* KO mice show an age-dependent neurodegenerative phenotype, central and peripheral axonal degeneration, reduced myelin volume and loss of axo-glial junctions. This phenotype was rescued by *B4GALNT1* expression in neurons but not by *B4GALNT1* glial expression, indicating that neuronal rather that glial gangliosides are important for integrity of the CNS (Yao et al. 2014). On the other hand, *SPG5* encodes an enzyme of the bilic acid and neurosteroid formation (CYP7B1). SPG5 physiopathology may result from an accumulation of toxic substrates and the absence of neurosteroids (Leoni and Caccia 2011; Theofilopoulos et al. 2014). In *Gba2* KO mice, the accumulation of glucocerebrosides has been shown in brain, liver and testis (Gonzalez-Carmona et al. 2012), but only infertility has been explored in these mice and whether this accumulation is toxic in the brain is not proved. Lipid storage may also be affected in some of these diseases since Atlastin-1, REEP1, Spartin and Seipin modulate lipid droplet structures (Renvoisé et al. 2012; Klemm et al. 2013; Falk et al. 2014).

Altogether, the functional knowledge gained from the known functions of the HSP genes and from the analysis of various animal models of these diseases suggest that the pathology results from disturbance of intracellular membrane trafficking and may account for the ‘dying-back’ mechanism observed in neuropathological human cases (Supplementary Table 2) (Deluca et al. 2004). The relationship between most of the genes involved in HSP has recently been pinpointed by the publication of an HSP interactome that may be useful to incriminate further causative genes in the future (Novarino et al. 2014). Very recently, nucleotide metabolism and autophagy were also novel functions reported as possibly altered in HSP (Oz-Levi et al. 2012; Novarino et al. 2014).

## Conclusions

The increasing number of genes and the extension of the clinical picture associated with each genetic entity are building a new nosology that may modify the way molecular diagnosis and treatment of these diseases is done. Although it is often impossible to identify the mutated HSP gene in an individual patient on the basis of clinical criteria, the relative frequencies and clinical characteristics still help to elaborate an effective diagnostic strategy after careful exclusion of other causes. The strategy is simple in certain cases because of the high relative frequencies of two major genes: SPG4, which represents up to 40 % of pure AD forms, and SPG11, which accounts for up to 59 % of cases with a TCC transmitted in an AR manner. However, numerous studies have shown that the classic subdivision of HSP into pure and complex forms, still in use in clinical practice, is imperfect. In addition, the clinical and genetic overlap of various neurodegenerative diseases suggests that HSP is (or indeed the HSPs are) in a continuum with other neurological diseases and that the phenotype of a given patient will depend on multiple factors, including the mutated gene, the nature of the mutation and its location in the protein, the zygosity of the mutation, modifier variants and the environment. When considering all factors, it becomes clear that the dogma linking one gene to one phenotype has to be replaced by one patient—one disease, which will fit with personalized medicine in the future. On the other hand, there is indication for a possible unification of genetic forms from the cell biological and, thus, potentially therapeutic point of view. In particular, the functions of several recently identified HSP proteins suggest that they may participate in the same molecular pathway of lipid metabolism, which may lead to common therapies. Interestingly, disturbances in lipid metabolism also offered the unprecedented opportunity to identify biomarkers for HSP, as in SPG5 (25 and 27 hydroxy-cholesterol) (Schüle et al. 2010) and SPG26 (testosterone or GM2/GM3 levels) (Boukhris et al. 2013; Harlalka et al. 2013) (Table 2; Supplementary Table 1), prerequisite for therapeutic trials. Finally, elucidation of the underlying pathogenic mechanisms will also help to develop more effective therapeutic agents. Preclinical trials in *Spast* KO flies and mice (ex vivo) and SPG4 human embryonic stem cells indicated that microtubule-binding agents might have therapeutic value (Fassier et al. 2013; Denton et al. 2014). Similarly, these drugs proved to be efficient in rescuing axon growth defects in SPG3A iPSC (Zhu et al. 2014) which open therapeutic avenues for HSP subtypes related to abnormal trafficking.

## Electronic supplementary material

Below is the link to the electronic supplementary material.

Supplementary material 1 (PDF 2642 kb)
