# Supplementary material for: Delving into the complexity of hereditary spastic paraplegias: how unexpected phenotypes and inheritance modes are revolutionizing their nosology
Source: Hum Genet. 2015 Mar 11;134(6):511–38. doi: 10.1007/s00439-015-1536-7 (PMC4424374; doi:10.1007/s00439-015-1536-7)
Supplement: Supplementary file 1 — Supplementary material 1 (PDF 2642 kb) [file 439_2015_1536_MOESM1_ESM.pdf]

HUMAN GENETICS

Delving into the complexity of hereditary spastic paraplegias: how unexpected phenotypes and inheritance modes are revolutionizing their nosology

Christelle Tesson, Jeanette Koht, Giovanni Stevanin

C. Tesson, G. Stevanin (✉)
INSERM U1127, CNRS UMR7225, Sorbonne Universités UPMC Univ Paris 06 UMR_S1127, Institut du Cerveau et de la Moelle épinière, EPHE, F-75013, Paris
e-mail: giovanni.stevanin@upmc.fr

J. Koht
Department of Neurology, Drammen Hospital, Vestre Viken Health Trust, Drammen, Norway

G. Stevanin
APHP, Hôpital de la Pitié-Salpêtrière, Département de Génétique et Cytogénétique, F-75013, Paris, France

Corresponding author: Giovanni Stevanin, Institut du Cerveau et de la Moelle épinière, CHU Pitié-Salpêtrière, 47 bd de l'Hôpital, F-75013, Paris, email: giovanni.stevanin@upmc.fr

Supplementary Fig. 1: Molecular diagnosis strategy according to the phenotype and the mode of transmission

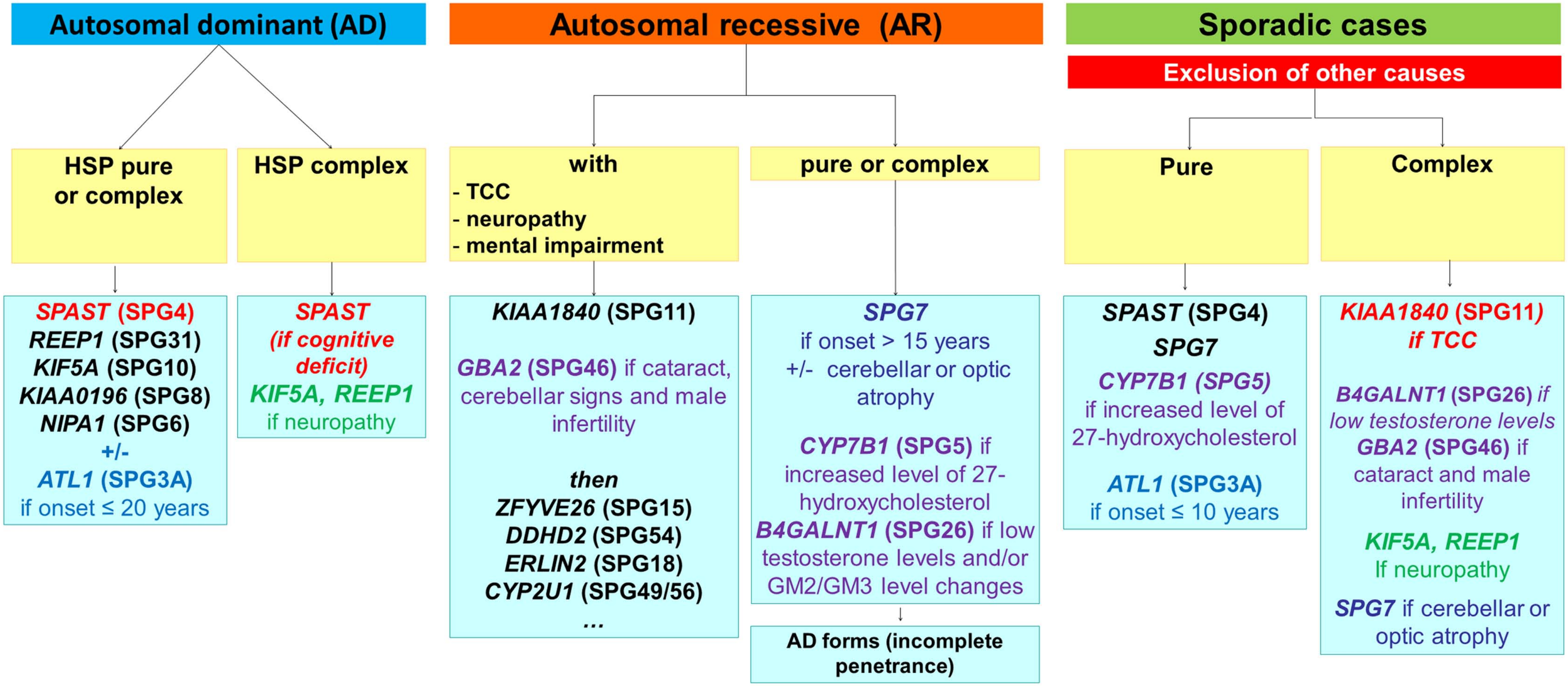

SUPPLEMENTARY TABLE 1: Syndromic forms with complex spastic paraplegia

Gene (OMIM n°)	Chromosome	Protein	Protein function	Inheritance	Key reference	Frequency / Number of families	Age at onset (y)	Associated clinical features and OMIM n°	MRI	Functional tests, biomarkers	Allelic disorders (OMIM n°)*	Treatment
ABCD1 300371	Xq28	ATP-binding cassette, subfamily D, member 1	Translocate a wide variety of substrates across extra- and intracellular membranes, including metabolic products, lipids, sterols and drugs	X linked	Mosser et al 1993<br>Guimaraes et al 2001<br>O'Neill et al 2001<br>Zhan et al 2013	5 families	9-42	Addison disease, adrenoleucodystrophy and spastic paraparesis with neurophysiological abnormalities or adrenomyeloneuropathy	Brain: Normal<br>Spinal cord: Altered	Increased of VLCFA in plasma and reduced beta-oxydation function	**Adrenoleukodystrophy (ALD):** childhood cerebral form, adrenomyeloneuropathy, adult cerebral, adolescent, adrenal insufficiency without neurologic disease, asymptomatic, and heterozygotes<br>300100	
ALDH3A2 609523	17p11	Aldehyde dehydrogenase family 3 subfamily A member 2	Oxydation of long chain aldehydes derived from lipid metabolism	AR	Rogers et al 1997	~100 families (8/100 000 in North Sweden Jagell et all 1981)	Infancy	**Sjögren-Larsson syndrome:** ichthyosis, MR, spastic paraparesis, macular dystrophy and leukoencephalopathy<br>Sjorgen and Larsson 1957<br>270200	Periventricular WMH and mild ventricular enlargement.	Elevated urinary concentration of LTB4 and 20-OH-LTB4<br>H-MR spectroscopy: abnormal white matter peak consistent with long-chain fatty alcohol accumulation		
ALS2 606352	2q33-q35	Alsin	Intracellular trafficking	AR	Eymard-Pierre et al 2002	9 families	1	Spastic tetraparesis, anarthria, dysphagia and slow eye movements.<br>607225	Normal or brainstem, spinal cord atrophy. Pyramidal tract hyperintensities.		**Amyotrophic lateral sclerosis**<br>205100<br>**Juvenile primary lateral sclerosis**<br>205100	
ARG1 608313	6q23	Arginase	Catalyses the last step of the urea cycle	AR	Haraguchi et al 1990	~30 families (1/1100000)	Infancy to 60	**Argininemia:** progressive mental impairment, spasticity, growth retardation and periodic episodes of hyperammonemia.<br>207800	ND	Elevation of plasma arginine level. No detectable arginase enzyme activity in red blood cell extracts.		Protein restriction
ARSA 607574	22q13	*Arylsulfatase A*	Lysosomal enzyme	AR	Polten etal 1991<br>Gieselmann et al 1991<br>Kondo et al 1991<br>Bohne et al 1991<br>Fluharty et al 1991	~10 families	13363	UL spasticity and psychiatric manifestations and sometimes optic atrophy, cognitive decline, frontal lobe type personality changes, polyneuropathy or epilepsy.	Leukodystrophy with symmetric WMH, cortical and subcortical atrophy	Positive urine sulfatide, low ASA activity	**Metachromatic leukodystrophy**<br>250100	
ARX 300382	Xq22	Aristalless-Related Homeobox, X-Linked	Homeodomain transcription factor	X linked	Kato et al 2004	~3 families	Birth	**Proud Syndrome:** corpus callosum agenesis, severe MR, seizures and spasticity	Corpus callosum agenesis			
ATN 607462	12p13	Atrophin 1	Transcription regulation	AD	Kasahata et al 2010	1 family	45	Case report: ataxia, spasticity of the right lower extremity and mild sensory disturbances	Dilatation of the 4th ventricle, mild atrophy of the superior cerebellar peduncles, mild atrophy of the cerebellum and atrophy of parietal lobes		**Dentatorubro-palladoluysian atrophy**<br>125370	
ATP6AP2 300556	Xp11.4	ATPase, H+ transporting lysosomal accessory protein 2	ATPase required for lysosomal degradation functions and autophagy	X linked	Korvatska et al 2013<br>Poorkaj et al 2010	1 family	14-50	Parkinsonism with spasticity<br>300911	Normal or mild diffuse atrophy and enlargement of 3rd ventricle.		MR and general tonico-clonic seizures<br>300423	
ATRX 300032	Xq13	ATRX-gene	Gene expression	X linked	Gibbons et al 1995<br>Lossi et al 1999	1 family	Birth	MR-hypotonic facies syndrome: severe MR, spasticity<br>309580	Normal			
ATXN3 607047	14q32.31	Ataxin 3	Component of ubiquitin proteasome system	AD	Wang et al 2009	6 families	12 to 48	Spasticity of LL, nystagmus, dysphagia and dysarthria	Normal		**Machado-Joseph disease:** ataxia, spasticity and ocular movement abnormalities.<br>109150	
CYP27A1 606530	2q33	Sterol 27-hydroxylase	Mitochondrial P450 cytochrome. Hydroxylation of sterol at C27 position.	AR	Cali et al 1991	~30 families (1/1100000)	Infancy	**Cerebrotendinous xanthomathosis:** deposition of cholesterol and cholesthanol in multiple tissues. Progressive neurological dysfunction (cerebellar ataxia, spinal cord involvement and pseudobulbar sign), tendon xanthomas, artherosclerosis and cataract.	Hyperintensity of the dentate nuclei, periventricular leukoencephalopathy, TCC cerebral atrophy.			Chenodeoxycholic acid
DARS 603084	12q21	Aspartyl tRNA synthetase	Aminoacylation of cognate tRNA in the initial step of ribosome-dependent protein biosynthesis	AR	Taft et al 2013	~10 families	Infancy	**Leukoencephalopathy:** hypomyelination with brainstem and spinal cord involvement and leg spasticity<br>615281	WMH			
ELOVL4 605512	6q14	Elongation of very long chain fatty acids-like 4	Elongation of fatty acid chains	AR	Zhang et al 2001	2 families	Birth	Ichthyosis, spastic quadriplegia, MR and seizures<br>Aldahmesh et al 2011<br>614457	Delayed myelination and brain atrophy		**Macular dystrophy (Stargardt's disease)**<br>AD 600110<br>**Spinocerebellar ataxia 34**<br>AD 133190	
FXN 606829	9q13	Frataxin	Mitochondrial chaperone involved in rion-sulfur biogenesis and heme biogenesis	AR	Delatycki et al 1999	1 family	Adulthood	Case report: lower limb spasticity at first, then of the upper limbs, dysarthria, dysphagia.	Normal		Friedreich ataxia<br>229300	
GAD1 605363	2q31.1	Glutamate Decarboxylase 1	Catalyses the conversion of glutamate into GABA	AR	Lynex et al 2004	1 family	1	Spastic cerebral palsy with LL spasticity, MR, hypertonia and ataxia<br>603513				

GALC<br>*606890*	14q31	Galactosylceramidase	Lysosomal enzyme involved in the catabolism of galactosylceramide	AR	Sakai et al 1994	~50 families	6 month-23 years	**Krabbe disease:** severe form that manifests at 6 months with irritability, spasticity and developmental delay. Death occurs before 2 years. Later onset patient may have spastic paraparesis only or with vision loss and intellectual regression<br>245200	Brain white matter demyelination			
GFAP<br>*137780*	17q21	Glial Fibrillary Acidic Protein	Intermediate filament	AD	Brenner et al 2001<br>Sawaishi et al 2002<br>Namekawa et al 2002	~20 families	Birth-33	**Alexander's disease:** seizures, megalencephaly, cognitive decline and spasticity.<br>203450	WMH			
GJA1	6q22	Gap junction protein, alpha 1	Connexin 43	AD/AR	Paznekas et al 2003	~20 families	11 to 60	**Oculodentodigitale dysplasia:** syndactyly of the fourth and fifth fingers, spastic paraparesis, earing loss, decreased visual acuity, ataxic gait<br>AD 164200 / AR 257850	WMH		**Atrioventricular septal defect (type 3)**<br>AR 600309<br>**Craniometaphyseal dysplasia**<br>AR 218400<br>**Hypoplastic left heart syndrome**<br>251550<br>**Syndactyly**<br>186100	
LMNB1<br>*150340*	5q31	Lamin B1	Nuclear lamina	AD	Padiath et al 2006	~13 families	40-50	**Adult demyelinating leukodystrophy** autonomic abnormalities, pyramidal and cerebellar dysfunction<br>169500	Hyperintensity of cortico-spinal tracts and cerebellar peduncles. WMH in the frontoparietal and periventricular area			
MECP2<br>*300005*	Xq28	Methyl-CpG-Binding Protein 2	Chromatin associated protein (Transcription activator and repressor)	X linked	Lindsay et al 1996<br>Meloni et al 2000<br>Lubs et al 1999<br>Meins et al 2005	~30 families (1/1100000)	Infancy	Spasticity with MR and sometimes autistic like features or tremor<br>300055<br>**Lubs MR:** MR, infantile hypotonia, mild dysmorphic features, poor speech development, autistic features, seizures, progressive spasticity, and recurrent infections.<br>300260	Normal		**Angelmann syndrome:** MR, movement disorders, abnormal behaviours, and severe limitations in speech and language.<br>105830<br>**Neonate encephalopathy:** microcephaly, short stature, hypotonia and seizures.<br>300673<br>**Rett syndrome:** delayed development between 6 and 18 months , regression of acquired skills, loss of speech, stereotypic movements, microcephaly, seizures, and MR<br>312750<br>**Autism susceptibility**<br>300496	
MTHFR	1p36	5,10-Methylenetetrahydrofolate reductase	Catalyses the conversion of 5,10-methylenetetrahydrofolate to 5-methyltetrahydrofolate, a cosubstrate for homocysteine remethylation to methionine	AR	Goyette et al 1994<br>Bathgate et al 2012	1 family	Young adult	Spastic paraplegia with seizures, frontal/subcortical deficit and cognitive decline	Cortical atrophy	Elevated homocysteine plasma levels	**Homocystinuria:** spectrum ranges from severe neurologic deterioration and early death to asymptomatic form in adults<br>236250	Folic acid, betaine, vitamin B12, riboflavin
PAH<br>*612349*	12q24	Phenylalanine hydroxylase	Catalyses the hydroxylation of phenylalanine to tyrosine	AR	Eisensmith and Woo 1992<br>Shimaru et al 1993<br>Weglage et al 2000<br>Kasim et al 2001	3 families	30-50	Case report: Spastic paraplegia with cognitive decline, ataxia and tremor	Normal or WMH	Elevated phenylalanine plasma concentration	**Phenylketonuria**<br>AR 261600	Low phenylalanine diet
PSEN1<br>*104311*	14q24	Presenilin 1	Gamma secretase component involved in APP metabolism	AD	Sherrington et al 1995<br>Crook et al 1998	6 families	28-57	Alzheimer's disease with spastic paraparesis and apraxia or unusual plaques<br>607822			**Alzheimer's disease**<br>AD 607822<br>**Cardiomyopathy, dilated**<br>AD 61369<br>**Frontotemporal dementia**<br>AD 600274<br>**Pick's disease**<br>AD 172700	
SETX<br>*608465*	9q34	Senataxin		AR	Moreira et al 2004<br>De Joughe et al 2002	3 families	4-49 years	Motor neuropathy with spastic paraplegia	Diffuse white matter lesions.	Increased plasmatic AFP	**Ataxia ocular apraxia 2:** ataxia with distal amyotrophy and oculomotor apraxia.<br>608465<br>**Amyotrophic lateral sclerosis 4**<br>602433	
SLC25A15<br>*603861*	13q14	Solute carrier family 25 (mitochondrial carrier, ornithine transporter), member 15	Ornithine transport across the inner mitochondrial membrane (Step of the urea cycle)	AR	Camacho et al 1999	~10 families	Neonatal-Infancy	**Hyperornithinemia-hyperammonemia-homocitrullinuria (HHH):** sometimes associated with MR, seizures, ataxia.<br>238970	Brain atrophy, basal ganglia calcification			Protein restriction, consider ornithine, arginine or citrulline supplementation

SLC2A1 *138140*	1p34	Solute carrier family 2, member 1	Major brain glucose transporter	AD	Weber et al 2011	~20 families	1-15	**Dystonia type 9:** paroxysmal choreathetosis and spastic paraparesis sometimes cognitive impairment, seizure and ataxia. 601042 **GLUT1 deficiency syndrome 1 and 2:** epileptic encephalopathy, delayed development, microcephaly, motor incoordination and spasticity. 606777 / 612126	ND		**Susceptibility to idiopathic generalized epilepsy** 614847	
SLC30A10 *611146*	1q41	Solute carrier family 30, member 10	Cellular efflux of zinc. Mn transporter (protects cell from Mn toxicity)	AR	Tsuchl et al 2012 Grospe et al 2000	1 family	Infancy	Polycythaemia and spastic paraparesis	High signal in the lenticulate and dentate nuclei, brainstem and pituitary consistent with manganese deposition. Brainstem atrophy		**Hypermanganesemia** with dystonia, polycysthaemia and cirrhosis 613280	
	11q13			AR	Macedo-Souza et al 2005	2 families	1	**SPOAN:** Optic atrophy, peripheral motosensory neuropathy. 609541				
	4q34.3-q35.2			AD	Dyck et al 1968	~20 families	<20	Hereditary motor and sensory neuropathy with spastic paraplegia (HMSN5) 600361				

* Inheritance mode is indicated when it differs from the one described in families with spasticity

AD=autosomal dominant, AFP=alpha-foeto-protein, AR=autosomal recessive, LL=lower limb, MR=mental retardation, MRI=magnetic resonance imaging, TCC=thin corpus callosum, UL=upper limb, VLCFA=very long chain of fatty acids, WMH=white matter hyperintensity, y=years

SUPPLEMENTARY TABLE 2: Main anatomopathological features according to the mutated gene

SPG n°	*Gene*	Observations					
		**Mutation**	**Clinical features**	**Morphological anomalies**	**Cellular abnormalities**	**Inclusions/ aggregates**	**Reference**
SPG2	*PLP1*	Y263C	Spastic paraplegia with dementia	Moderate atrophy of the cerebellum with ventricular dilatation and softening of the white matter throughout the CNS. Myelin pallor of several tractus and severe axonal degeneration of corticospinal tracts			Suzuki et al., 2011
SPG4	*SPAST*	A1395G	Pure spastic paraplegia	Cortico spinal tract pathology most pronounced in the dorsal and lumbar spinal cord. Loss of neurons in the substantia nigra and hippoccampus		Lewy bodies ubiquitin and α-synuclein positives in the substantia nigra. Neurofibrillary tangles tau positive in hippoccampus. Granuloavacuolar degeneration, tau and α-β-crystallin inclusions in limbic structures and neocortex	White et al., 2000
SPG6	*NIPA1*	G106R		Distal axonal degeneration of corticospinal tracts and dorsal columns with myelin loss associated with motor neuron loss.	Granular polarized cytoplasmic labeling in neurons of frontal cortex and spinal cord. NIPA1 expression appeared decreased in frontal cortex	TDP-43 positive neuronal cytoplasmic inclusions in lower motor neurons, substantia nigra, basal ganglia, limbic structures and neocortex. Bunina bodies in motor neurons	Martinez-Lage et al., 2012
SPG11	*KIAA1840*	L733X	Juvenile amyotrophic lateral sclerosis and long time survival (El Escorial criteria)	No cerebral or cerebellar atrophy, normal corpus callosum, no myelinic pallor. Atrophy of spinal cord anterior horn and myelinic pallor in the anterolateral columns. Motor neuron loss. Neurogenic muscular atrophy	Central chromatolysis	Pigmentary neurodegeneration, hyaline inclusions	Orlacchio et al., 2010
SPG43	*C19orf12*	G53R	**Neurodegeneration with brain iron accumulation-like disorder :** optic atrophy, spastic tetraparesis, ataxia, marked dysarthria, axonal motor neuropathy and cognitive decline.	Loss of myelin in pyramidal and optic tracts		Iron deposition in globus pallidus and substantia nigra<br>Ubiquitin positive axonal speroids in globus pallidus, putamen and caudate nucleus<br>Alphasynuclein deposits in neurites and lewy body<br>Hyperphosphorylated tau neuronal inclusions	Hartig et al., 2011
SPG50	*AP4M1*	c.1137+1G>T	**Cerebral Palsy:** tetraplegic cerebral palsy with mental retardation, reduction of cerebral white matter and cerebellar atrophy.	Widening of ventricules, severe thinning of the corpus callosum, large edema in the cortex and basal ganglia causing a spongiform apparence, pontine atrophy, thining of the cerebellar granular layer neuron loss of dentate nucleus	Shrunken neurons in cortex and basal ganglia. Purkinje cell atrophy and decreased dendritic arborisation	Aberrant GluRδ2 glutamate receptor localization and abnormal dendritic spine morphology	Verkek et al., 2009

ATP6AP2	*ATP6AP2*	S115S (splicing modification)	Spastic paraplegia with parkinsonism features	Mild ventriculomegaly, neuronal loss in the substantia nigra with rare neurofibrillary tangles	Reduced ATP6AP2 brain immunostaining, astrocytic tau immunostaining	Tau positive neurofibrillary tangles in temporal lobe, diffuse Aβ deposits in neocortex and limbic system. Massive accumulation of p62/SQTM1	Poorkaj et al., 2010 Korvatsaka et al., 2013
GFAP	*GFAP*	R276L	Alexander's Disease : Tetraparesis with dysarthria and dysphagia	Severe lesions of bilateral pyramides of the medulla oblongata, degeneration with astrogliosis and macrophage infiltration in the lateral and anterior corticospinal tracts		Presence of rosenthal fibers ubiquitin, β-cristallin and GFAP-positive in various structures	Namekawa et al., 2002

References

Bathgate D, Yu-Wai-Man P, Webb B, et al (2012) Recessive spastic paraparesis associated with complex I deficiency due to MTHFR mutations. J Neurol Neurosurg Psychiatry 83:115.

Bohne W, von Figura K, Gieselmann V (1991) An 11-bp deletion in the arylsulfatase A gene of a patient with late infantile metachromatic leukodystrophy. Hum Genet 87:155–158.

Brenner M, Johnson AB, Boespflug-Tanguy O, et al (2001) Mutations in GFAP, encoding glial fibrillary acidic protein, are associated with Alexander disease. Nat Genet 27:117–120.

Cali JJ, Hsieh CL, Francke U, Russell DW (1991) Mutations in the bile acid biosynthetic enzyme sterol 27-hydroxylase underlie cerebrotendinous xanthomatosis. J Biol Chem 266:7779–7783.

Camacho JA, Obie C, Biery B, et al (1999) Hyperornithinaemia-hyperammonaemia-homocitrullinuria syndrome is caused by mutations in a gene encoding a mitochondrial ornithine transporter. Nat Genet 22:151–158.

Crook R, Verkkoniemi A, Perez-Tur J, et al (1998) A variant of Alzheimer’s disease with spastic paraparesis and unusual plaques due to deletion of exon 9 of presenilin 1. Nat Med 4:452–455.

De Jonghe P, Auer-Grumbach M, Irobi J, et al (2002) Autosomal dominant juvenile amyotrophic lateral sclerosis and distal hereditary motor neuronopathy with pyramidal tract signs: synonyms for the same disorder? Brain 125:1320–1325.

Delatycki MB, Knight M, Koenig M, et al (1999) G130V, a common FRDA point mutation, appears to have arisen from a common founder. Hum Genet 105:343–346.

Dyck PJ, Lambert EH (1968) Lower motor and primary sensory neuron diseases with peroneal muscular atrophy. II. Neurologic, genetic, and electrophysiologic findings in various neuronal degenerations. Arch Neurol 18:619–625.

Eisensmith RC, Woo SL (1992) Molecular basis of phenylketonuria and related hyperphenylalaninemias: mutations and polymorphisms in the human phenylalanine hydroxylase gene. Hum Mutat 1:13–23.

Eymard-Pierre E, Lesca G, Dollet S, et al (2002) Infantile-onset ascending hereditary spastic paralysis is associated with mutations in the alsin gene. Am J Hum Genet 71:518–527.

Fluharty AL, Fluharty CB, Bohne W, et al (1991) Two new arylsulfatase A (ARSA) mutations in a juvenile metachromatic leukodystrophy (MLD) patient. Am J Hum Genet 49:1340–1350.

Gibbons RJ, Picketts DJ, Villard L, Higgs DR (1995) Mutations in a putative global transcriptional regulator cause X-linked mental retardation with alpha-thalassemia (ATR-X syndrome). Cell 80:837–845.

Gieselmann V, Fluharty AL, Tønnesen T, Von Figura K (1991) Mutations in the arylsulfatase A pseudodeficiency allele causing metachromatic leukodystrophy. Am J Hum Genet 49:407–413.

Gospe SM Jr, Caruso RD, Clegg MS, et al (2000) Paraparesis, hypermanganesaemia, and polycythaemia: a novel presentation of cirrhosis. Arch Dis Child 83:439–442.

Goyette P, Sumner JS, Milos R, et al (1994) Human methylenetetrahydrofolate reductase: isolation of cDNA, mapping and mutation identification. Nat Genet 7:195–200.

Guimarães CP, Lemos M, Menezes I, et al (2001) Characterisation of two mutations in the ABCD1 gene leading to low levels of normal ALDP. Hum Genet 109:616–622.

Haraguchi Y, Aparicio JM, Takiguchi M, et al (1990) Molecular basis of argininemia. Identification of two discrete frame-shift deletions in the liver-type arginase gene. J Clin Invest 86:347–350.

Hartig MB, Iuso A, Haack T, et al (2011) Absence of an Orphan Mitochondrial Protein, C19orf12, Causes a Distinct Clinical Subtype of Neurodegeneration with Brain Iron Accumulation. Am J Hum Genet 89:543–550.

Ishimaru K, Tamasawa N, Baba M, et al (1993) Phenylketonuria with adult-onset neurological manifestation. Rinshō Shinkeigaku Clin Neurol 33:961–965.

Kasim S, Moo LR, Zschocke J, Jinnah HA (2001) Phenylketonuria presenting in adulthood as progressive spastic paraparesis with dementia. J Neurol Neurosurg Psychiatry 71:795–797.

Kasahata N, Iwasaki Y (2010) Dentatorubropallidoluysian atrophy without involuntary movement or dementia--a case report. Clin Neurol Neurosurg 112:722–725.

Kato M, Das S, Petras K, et al (2004) Mutations of ARX are associated with striking pleiotropy and consistent genotype-phenotype correlation. Hum Mutat 23:147–159.

Kondo R, Wakamatsu N, Yoshino H, et al (1991) Identification of a mutation in the arylsulfatase A gene of a patient with adult-type metachromatic leukodystrophy. Am J Hum Genet 48:971–978.

Korvatska O, Strand NS, Berndt JD, et al (2013) Altered splicing of ATP6AP2 causes X-linked parkinsonism with spasticity (XPDS). Hum Mol Genet 22:3259–3268.

Lindsay S, Splitt M, Edney S, et al (1996) PPM-X: a new X-linked mental retardation syndrome with psychosis, pyramidal signs, and macroorchidism maps to Xq28. Am J Hum Genet 58:1120–1126.

Lossi AM, Millan JM, Villard L, et al (1999) Mutation of the XNP/ATR-X gene in a family with severe mental retardation, spastic paraplegia and skewed pattern of X inactivation: demonstration that the mutation is involved in the inactivation bias. Am J Hum Genet 65:558–562.

Lubs H, Abidi F, Bier JA, et al (1999) XLMR syndrome characterized by multiple respiratory infections, hypertelorism, severe CNS deterioration and early death localizes to distal Xq28. Am J Med Genet 85:243–248.
Lynex CN, Carr IM, Leek JP, et al (2004) Homozygosity for a missense mutation in the 67 kDa isoform of glutamate decarboxylase in a family with autosomal recessive spastic cerebral palsy: parallels with Stiff-Person Syndrome and other movement disorders. BMC Neurol 4:20.
Macedo-Souza LI, Kok F, Santos S, et al (2005) Spastic paraplegia, optic atrophy, and neuropathy is linked to chromosome 11q13. Ann Neurol 57:730–737. doi: 10.1002/ana.20478
Martinez-Lage M, Molina-Porcel L, Falcone D, et al (2012) TDP-43 pathology in a case of hereditary spastic paraplegia with a NIPA1/SPG6 mutation. Acta Neuropathol (Berl) 124:285–291.
Meins M, Lehmann J, Gerresheim F, et al (2005) Submicroscopic duplication in Xq28 causes increased expression of the MECP2 gene in a boy with severe mental retardation and features of Rett syndrome. J Med Genet 42:e12.
Meloni I, Bruttini M, Longo I, et al (2000) A mutation in the rett syndrome gene, MECP2, causes X-linked mental retardation and progressive spasticity in males. Am J Hum Genet 67:982–985.
Moreira M-C, Klur S, Watanabe M, et al (2004) Senataxin, the ortholog of a yeast RNA helicase, is mutant in ataxia-ocular apraxia 2. Nat Genet 36:225–227.
Mosser J, Douar AM, Sarde CO, et al (1993) Putative X-linked adrenoleukodystrophy gene shares unexpected homology with ABC transporters. Nature 361:726–730.
Namekawa M, Takiyama Y, Aoki Y, et al (2002) Identification of GFAP gene mutation in hereditary adult-onset Alexander's disease. Ann Neurol 52:779–785.
O'Neill GN, Aoki M, Brown RH (2001) ABCD1 translation-initiator mutation demonstrates genotype-phenotype correlation for AMN. Neurology 57:1956–1962.
Orlacchio A, Babalini C, Borreca A, et al (2010) SPATACSIN mutations cause autosomal recessive juvenile amyotrophic lateral sclerosis. Brain 133:591–598.
Padiath QS, Saigoh K, Schiffmann R, et al (2006) Lamin B1 duplications cause autosomal dominant leukodystrophy. Nat Genet 38:1114–1123.
Paznekas WA, Boyadjiev SA, Shapiro RE, et al (2003) Connexin 43 (GJA1) mutations cause the pleiotropic phenotype of oculodentodigital dysplasia. Am J Hum Genet 72:408–418.
Polten A, Fluharty AL, Fluharty CB, et al (1991) Molecular basis of different forms of metachromatic leukodystrophy. N Engl J Med 324:18–22.
Poorkaj P, Raskind WH, Leverenz JB, et al (2010) A novel X-linked four-repeat tauopathy with Parkinsonism and spasticity. Mov Disord 25:1409–1417.
Rogers GR, Markova NG, De Laurenzi V, et al (1997) Genomic organization and expression of the human fatty aldehyde dehydrogenase gene (FALDH). Genomics 39:127–135.
Sawaishi Y, Yano T, Takaku I, Takada G (2002) Juvenile Alexander disease with a novel mutation in glial fibrillary acidic protein gene. Neurology 58:1541–1543.
Sherrington R, Rogaev EI, Liang Y, et al (1995) Cloning of a gene bearing missense mutations in early-onset familial Alzheimer's disease. Nature 375:754–760.
Suzuki SO, Iwaki T, Arakawa K, et al (2011) An autopsy case of adult-onset hereditary spastic paraplegia type 2 with a novel mutation in exon 7 of the proteolipid protein 1 gene. Acta Neuropathol (Berl) 122:775–781.
Taft RJ, Vanderver A, Leventer RJ, et al (2013) Mutations in DARS cause hypomyelination with brain stem and spinal cord involvement and leg spasticity. Am J Hum Genet 92:774–780.
Tuschl K, Clayton PT, Gospe SM, et al (2012) Syndrome of hepatic cirrhosis, dystonia, polycythemia, and hypermanganesemia caused by mutations in SLC30A10, a manganese transporter in man. Am J Hum Genet 90:457–466.
Verkerk AJMH, Schot R, Dumee B, et al (2009) Mutation in the AP4M1 gene provides a model for neuroaxonal injury in cerebral palsy. Am J Hum Genet 85:40–52.
Wang Y, Du J, Wang J, et al (2009) Six cases of SCA3/MJD patients that mimic hereditary spastic paraplegia in clinic. J Neurol Sci 285:121–124.
Weber YG, Kamm C, Suls A, et al (2011) Paroxysmal choreoathetosis/spasticity (DYT9) is caused by a GLUT1 defect. Neurology 77:959–964.
Weglage J, Oberwittler C, Marquardt T, et al (2000) Neurological deterioration in adult phenylketonuria. J Inherit Metab Dis 23:83–84.
White KD, Ince PG, Lusher M, et al (2000) Clinical and pathologic findings in hereditary spastic paraparesis with spastin mutation. Neurology 55:89–94.
Zhang K, Kniazeva M, Han M, et al (2001) A 5-bp deletion in ELOVL4 is associated with two related forms of autosomal dominant macular dystrophy. Nat Genet 27:89–93.
Zhan Z-X, Liao X-X, Du J, et al (2013) Exome sequencing released a case of X-linked adrenoleukodystrophy mimicking recessive hereditary spastic paraplegia. Eur J Med Genet 56:375–378.
